# Current Approaches and Emerging Strategies in the Treatment of Peritoneal Carcinomatosis

**DOI:** 10.3390/ijms27156623

**Published:** 2026-07-24

**Authors:** Anna Alyasova, Kanamat Efendiev, Igor Reshetov, Dinara Ilyasova, Olga Shpileva, Victor Loschenov, Vladimir Makarov, Evgenia Zakharova, Pavel Karalkin, Yulia Agakina, Aida Gilyadova, Vadim Cheremisov, Andrey Stetsiuk, Alim Nebezhev, Polina Kozlova, Aminat Ataeva, Ekaterina Rostislavova, Valeria Sudarkina, Artem Shiryaev

**Affiliations:** 1 Department of Oncology, Radiotherapy and Reconstructive Surgery, Levshin Institute of Cluster Oncology, Sechenov First Moscow State Medical University, 119435 Moscow, Russia; alyasova_a_v@staff.sechenov.ru (A.A.); reshetov_i_v@staff.sechenov.ru (I.R.); dr.dinara_batyrbekovna@mail.ru (D.I.); shpileva_o_v@staff.sechenov.ru (O.S.); karalkin_p_o@staff.sechenov.ru (P.K.); y.agakina@gmail.com (Y.A.); gilyadova_a_v@staff.sechenov.ru (A.G.); cheremisov_v_v@staff.sechenov.ru (V.C.); drstetsyuk@mail.ru (A.S.); alim-nebezhev@mail.ru (A.N.); kozlova_p_e@staff.sechenov.ru (P.K.); ataevaamina002@gmail.com (A.A.); rostislavova_e_a@student.sechenov.ru (E.R.); sudarkina_v_p@student.sechenov.ru (V.S.); artemdoc@mail.ru (A.S.); 2 Department of Oncology, Radiation Therapy and Radiology, Privolzhsky Research Medical University, 603005 Nizhny Novgorod, Russia; ispclinic@mail.ru; 3 Department of Medical Photonics, Prokhorov General Physics Institute, Russian Academy of Sciences, 119991 Moscow, Russia; loschenov@mail.ru (V.L.); vi.makarov@physics.msu.ru (V.M.); 4 Department of Laser Micro-, Nano-, and Biotechnology, Institute of Engineering Physics for Biomedicine, National Research Nuclear University “MEPhI”, 115409 Moscow, Russia; 5 Institute of Mathematics and Natural Sciences, Kabardino-Balkarian State University, 360004 Nalchik, Russia; 6 Laboratory of Neurobiology and Tissue Engineering, Brain Science Institute, Research Center of Neurology, 125367 Moscow, Russia

**Keywords:** peritoneal carcinomatosis, cytoreductive surgery, hyperthermic intraperitoneal chemotherapy, pressurized intraperitoneal aerosol chemotherapy, electrostatic pressurized intraperitoneal aerosol chemotherapy, photodynamic therapy, photothermal therapy, nanomedicine, immune checkpoint inhibitors, chemoresistance

## Abstract

Peritoneal carcinomatosis (PC) is a common, prognostically unfavorable manifestation of advanced gastrointestinal and gynecological malignancies whose treatment is constrained by the blood-peritoneal barrier, which limits drug penetration even when cytoreductive surgery is combined with systemic chemotherapy. This review evaluates current intraperitoneal treatment modalities and emerging technologies for overcoming this limitation, based on recent clinical trials and meta-analyses identified through Scopus and PubMed. We sequentially examine conventional intraperitoneal chemotherapy (IPC); hyperthermic intraperitoneal chemotherapy (HIPEC) in ovarian, gastric, and colorectal cancers, with attention to patient selection and the peritoneal cancer index as determinants of survival benefit; pressurized intraperitoneal aerosol chemotherapy (PIPAC) and its electrostatic precipitation variant (ePIPAC); and photodynamic and photothermal therapy for tumor treatment. Novel strategies are also discussed, including IPC combined with immune checkpoint inhibitors, optical dosimetry, and nanomedicine-based drug delivery as tools for improving treatment precision. Overall, management of PC is transitioning toward a personalized, multimodal approach: HIPEC and PIPAC provide standardized platforms for regional therapy, but future progress depends on integrating advanced drug delivery systems, immunotherapy, and real-time intraoperative monitoring, with standardization of protocols through large multicenter trials remaining a priority for translating these modalities into clinical practice.

## 1. Introduction

Peritoneal carcinomatosis (PC) is a malignant involvement of the serous membrane lining the abdominal cavity and covering the visceral organs, which in most cases results from the progression of ovarian and gastrointestinal malignancies. PC is detected in 70–80% of patients with ovarian cancer (OC) at the time of diagnosis [1,2], a factor that significantly worsens the prognosis. In patients with colorectal cancer (CRC), the peritoneum represents the second most common site of metastatic spread. PC is identified in nearly 10% of CRC cases at initial diagnosis and in 20–50% of patients with recurrent disease [3]. Peritoneal metastases are detected in approximately half of all patients with recurrent gastric cancer (GC).

In addition, PC may represent a primary tumor entity, including peritoneal mesothelioma, primary peritoneal carcinoma, and pseudomyxoma peritonei. More rarely, in up to 10% of cases, PC results from the progression of malignancies located outside the abdominal cavity, such as lung or breast cancer, whereas in 3–5% of cases, its origin remains unknown [4]. In a large proportion of cases, PC is not accompanied by systemic dissemination of disease [5], indicating its locoregional nature. PC is also frequently detected incidentally during surgery.

A major obstacle to the effective treatment of PC is the blood-peritoneal barrier, which restricts the penetration of systemically administered cytotoxic agents into peritoneal tumor nodules and limits drug concentrations at the tumor site to levels well below those required for cytotoxic effect. Because the peritoneal cavity is relatively isolated from the systemic circulation, direct intraperitoneal drug delivery can, in principle, achieve substantially higher local drug concentrations than systemic administration while limiting systemic exposure and toxicity. This pharmacokinetic rationale underlies the development of IPC, HIPEC, and PIPAC, as well as newer locoregional approaches such as photodynamic (PDT) and photothermal (PTT) therapy, all of which aim to overcome the limitations imposed by the blood-peritoneal barrier and by impaired drug penetration into poorly vascularized tumor nodules.

Cytoreductive surgery (CRS) remains the cornerstone of treatment aimed at maximal tumor debulking. However, even after radical surgery, micrometastatic foci may persist—as demonstrated in 60–75% of OC cases—according to contemporary immunohistochemical and molecular analyses [6]. Overall, the prognosis of PC remains poor, as patient survival is typically less than 20 months despite treatment. The objective of this review is to provide a comprehensive and structured overview of the current therapeutic landscape and future perspectives in the management of PC. We systematically analyze the clinical data, meta-analyses, and trial outcomes establishing the roles of chemotherapy, PDT and PTT in OC, GC, and CRC.

Several reviews have addressed HIPEC or PIPAC in isolation, or have focused on a single tumor type; to our knowledge, none has systematically integrated conventional IPC, HIPEC, PIPAC/ePIPAC, PDT and PTT, and emerging nanomedicine- and immunotherapy-based strategies within a single comparative framework across OC, GC, and CRC. This review, therefore, differs from prior publications in three respects. First, it prioritizes randomized controlled trials and meta-analyses published between 2020 and 2026, capturing recent trial results not covered by earlier reviews. Second, it explicitly contrasts patient-selection criteria and outcomes across the three principal tumor types rather than treating PC as a single homogeneous entity. Third, it positions photodynamic and photothermal modalities, as well as nanomedicine-based delivery, not as isolated experimental techniques, but as complementary components of an emerging multimodal, personalized treatment strategy for PC, and critically discusses the translational barriers that currently separate these approaches from clinical practice.

## 2. Intraperitoneal Chemotherapy

In patients with advanced OC, first-line platinum-based therapy induces a response in approximately 70% of cases; however, disease recurrence subsequently develops in up to 95% of patients and may prove to be platinum-resistant [7]. When recurrence occurs within 6 months after completion of prior treatment, the response rate to a new course of chemotherapy does not exceed 15%, progression-free survival (PFS) is short, and median overall survival (OS) is less than 1 year [8]. Moreover, with each subsequent relapse, the likelihood of platinum resistance increases. The addition of bevacizumab to chemotherapy or the use of PARP inhibitors in patients with germline BRCA1/2 mutations, which are detected in 15–20% of cases, does not fully resolve the problem of micrometastatic disease and prevention of OC recurrence. Over the past three decades, the 5-year survival rate after treatment has remained below 40%, underscoring the need to search for new therapeutic options for this disease [9].

Among strategies aimed at improving recurrence-free survival (RFS) in patients with OC, the use of intraperitoneal chemotherapy (IPC) has been considered. Administration of a drug into the abdominal cavity provides increased intratumoral concentrations while reducing systemic absorption. This treatment modality may be repeated and combined with systemic chemotherapy. In one of the earliest studies, conducted by D.K. Armstrong et al. (2006) in patients with OC, median PFS and median OS were significantly higher in the IPC group than in the systemic therapy group (*p* = 0.05 and *p* = 0.03, respectively, by the log-rank test) [10]. However, subsequent studies evaluating the efficacy of IPC in patients with OC have not confirmed these findings in phase III trials, and the currently available data on the use of IPC remain insufficient [11].

The available literature also contains reports of the benefits of intraperitoneal paclitaxel administration in patients with GC in terms of median OS (*p* = 0.080 by the log-rank test) and an increased 3-year survival rate (21.9% vs. 6.0%) compared with systemic administration of the drug [12]. In an open-label, single-center, single-arm phase II trial (NCT05204173), the feasibility of adding a PD-1 inhibitor (sintilimab) to the “triplet” regimen consisting of intraperitoneal and intravenous paclitaxel plus oral S-1 was investigated. The survival outcomes obtained (median OS, 18.4 months; median PFS, 14.6 months; objective response rate, 57.9%) exceeded those of historical controls, thereby rendering this combination a promising candidate for first-line therapy in patients with GC and PC [13]. However, the single-arm design, small sample size, lack of external validity, statistical limitations, and the potentially high cumulative toxicity of the agents employed necessitate further investigation. Several clinical trials are currently investigating combinations of IPC with systemic administration of anticancer agents [11]. A meta-analysis conducted by Al Mahrizi et al. (2026), which included seven studies (three randomized controlled trials and four cohort studies; *n* = 1264), evaluated adult patients who underwent radical resection of CRC followed by intraperitoneal administration of platinum-containing agents, as compared with a control group [14]. Platinum-based intraperitoneal therapy improved PFS (*p* = 0.0004), showed a trend toward improved OS (*p* = 0.28), and was associated with a reduced rate of peritoneal recurrence, although substantial heterogeneity was observed (*p* = 0.40) [14]. No increase in grade 3–4 adverse events or treatment-related complications was reported. Palliative IPC in patients with CRC is also still under investigation in clinical trials [15].

In addition to potential complications related to catheter placement, the disadvantages of IPC include treatment-related toxicity [16]; moreover, there are reports indicating that more than 50% of patients were unable to complete the planned treatment cycles [10]. More recent studies, however, have reported improved tolerability of IPC [12]. Furthermore, insufficient intratumoral penetration and nonuniform distribution of therapeutic agents remain important limitations [8]. IPC is usually performed only in patients who have undergone optimal CRS, several weeks after the operation, when adhesions have already formed and may impair distribution of the solution throughout the abdominal cavity. IPC has not been incorporated into routine clinical practice because [17]:

- there is no convincing evidence linking high local (peritoneal) drug exposure with improved intratumoral drug concentrations and increased OS;
- substantial heterogeneity exists across studies in dosing regimens, chemotherapeutic agents, and tumor types;
- the fact that most studies have been conducted in Asia may significantly affect outcomes such as OS when these data are applied to Western populations.

Collectively, these limitations of systemic chemotherapy and IPC define the rationale for the locoregional approaches discussed in this review. Systemic chemotherapy is fundamentally constrained by the blood-peritoneal barrier, which caps achievable intratumoral drug concentrations regardless of dose intensification. IPC partially circumvents this barrier by direct instillation but does not resolve the two problems that most limit its efficacy: shallow drug penetration into poorly vascularized tumor nodules and non-uniform distribution across the peritoneal surface, the latter being exacerbated by postoperative adhesions when IPC is delayed after CRS. HIPEC was developed specifically to address both problems simultaneously: performing perfusion intraoperatively, before adhesions form, improves distribution, while hyperthermia increases drug penetration and exerts direct thermal cytotoxicity and chemosensitization. PIPAC, in turn, was developed for patients with inoperable or unresectable disease where HIPEC is not feasible, using pressurized aerosolization to improve drug distribution and tissue uptake at substantially lower systemic drug doses than IPC or HIPEC.

## 3. Hyperthermic Intraperitoneal Chemotherapy

Hyperthermic intraperitoneal chemotherapy (HIPEC) is an innovative treatment modality for advanced intra-abdominal malignancies that involves the intraperitoneal administration of cytotoxic agents heated to 41–43 °C. Unlike IPC, HIPEC is performed immediately after CRS, before adhesions form, thereby ensuring more uniform distribution of chemotherapeutic agents, while the thermal effect of hyperthermia enhances drug penetration into tumor foci. HIPEC also offers several additional advantages over IPC:

- It eliminates the need for devices providing postoperative peritoneal access and therefore avoids complications associated with catheter placement [18];
- Hyperthermia exerts direct cytotoxic activity against tumor cells and acts synergistically with many anticancer agents [19];
- It increases the sensitivity of tumor cells to drug therapy [20];
- It enhances the antitumor immune response, increases lymphocyte migration, and activates antigen-presenting cells [21].

**Figure 1:**
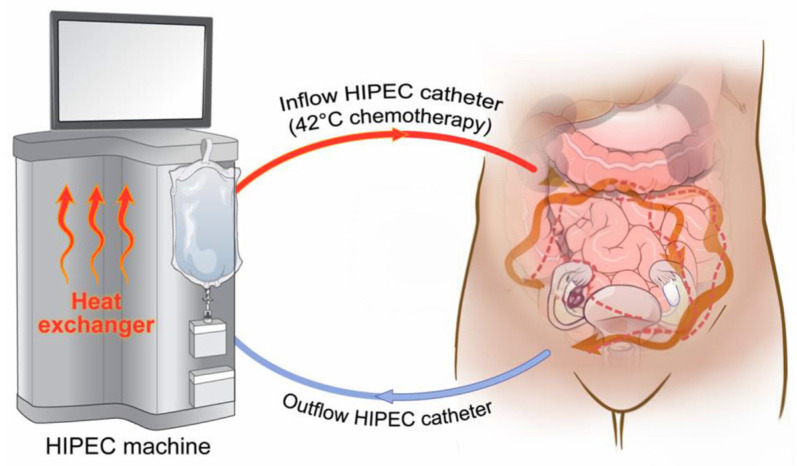
Schematic representation of the concept of hyperthermic intraperitoneal chemotherapy (HIPEC) in a patient with peritoneal carcinomatosis. Heated chemotherapeutic solution (42 °C) is circulated through the abdominal cavity via an inflow HIPEC catheter, distributed across the peritoneal surfaces, and recovered through an outflow catheter; the closed-circuit system maintains the target temperature using a heat exchanger within the HIPEC machine. Adapted from [22].

The concept of HIPEC and the underlying principles of regional drug delivery during the procedure are illustrated in Figure 1.

However, the depth of penetration of chemotherapeutic agents into tumor tissue during HIPEC does not exceed 1–5 mm, and uniform distribution of the drug is difficult to achieve because of the anatomical configuration of the abdominal cavity [23]. In addition, a major limitation of this method remains its toxicity, which may lead to patient death [24], thereby restricting the use of HIPEC in patients with initially reduced functional reserves [25].

### 3.1. Ovarian Cancer

More than a dozen international randomized phase III trials investigating the use of HIPEC in patients with OC have been registered [8]. For example, the completed randomized phase III OVHIPEC-1 trial (NCT00426257) [26] demonstrated that the addition of HIPEC to CRS in patients with stage III epithelial OC receiving neoadjuvant therapy increased median RFS (*p* < 0.001) and OS (*p* = 0.011) compared with the standard treatment group. In the randomized trial NCT01091636 conducted in South Korea, patients with stage III or IV OC and residual tumor burden less than 1 cm after CRS underwent HIPEC. Statistically significant differences in median RFS (*p* = 0.04) and OS (*p* = 0.04) compared with the control group were identified in the subgroup of patients who underwent CRS and HIPEC after neoadjuvant chemotherapy, but not in women who received HIPEC after primary CRS (*p* = 0.43 and *p* = 0.52, respectively) [27]. However, these findings are based on an unplanned subgroup analysis and therefore cannot be regarded as definitive evidence. Overall, the study failed to demonstrate the efficacy of HIPEC in the general population of patients with advanced OC. A comparative analysis of these two studies (OVHIPEC-1 and NCT01091636) [26,27] convincingly indicates that a clinically meaningful benefit from HIPEC is observed only after neoadjuvant chemotherapy, which appears to be related to the potentiating effect of hyperthermia on cisplatin cytotoxicity, but only in cells already “primed” by prior chemotherapy for a second therapeutic insult. When performed at the time of primary surgery, HIPEC may, on the contrary, worsen treatment outcomes. The methodological advantages of OVHIPEC-1, including a prespecified analysis of outcomes, longer follow-up (10 years versus 5.8 years in NCT01091636), and a higher cisplatin dose (100 mg/m^2^ versus 75 mg/m^2^), render the results of this trial more relevant to clinical practice.

According to the results of the multicenter open-label randomized phase III CHIPOR trial [28], the addition of HIPEC to CRS after response to platinum-based chemotherapy at first recurrence of epithelial OC significantly improved OS by 8.5 months (*p* = 0.024), with a 27% reduction in the risk of death. Use of HIPEC was associated with grade 3 or higher toxic adverse events 1.8 times more frequently than in patients who did not receive this treatment (49% vs. 27%, respectively). However, the 60-day postoperative mortality rate in the HIPEC group was zero, whereas three deaths were recorded in the control group. The findings reported in the CHIPOR trial define the indications for HIPEC at recurrence in a highly specific subgroup of patients with OC, namely those with a first platinum-sensitive recurrence, high-grade serous or endometrioid adenocarcinoma, and receipt of six cycles of platinum-based chemotherapy prior to surgery. The limitations of the study include its unconventional design, involving six cycles of chemotherapy before CRS, the absence of standardized preoperative patient selection, the unclear relationship between the improvement in OS and the lack of a clinically meaningful improvement in PFS (only +0.7 months), the absence of a direct comparison group, as well as the lack of information on key prognostic factors characterizing the disease, such as residual tumor burden after primary surgery. Taken together, the results of the three aforementioned studies allow, to a certain extent, a more personalized approach to the use of HIPEC in patients with OC: the absence of indications for HIPEC after primary cytoreduction, the possibility of performing the procedure after neoadjuvant chemotherapy and interval cytoreduction, and its potential role at recurrence following preoperative chemotherapy. However, incorporation of this method into routine clinical practice requires additional high-quality studies.

A subsequent randomized phase III trial, HORSE (MITO-18), directly tested whether HIPEC adds benefit to secondary cytoreductive surgery (SCRS) in platinum-sensitive recurrent OC, but, unlike CHIPOR, enrolled patients who had received no chemotherapy before surgery [29]. Among 167 patients (82 to SCRS + HIPEC, 85 to SCRS alone) with a median platinum-free interval of 18 months and a 95% complete-resection rate, the addition of cisplatin-based HIPEC (75 mg/m^2^ for 60 min at 41.5 °C) did not improve median PFS (25 vs. 23 months; HR 1.02, 95% CI 0.73–1.42; *p* = 0.91), nor did it improve postrecurrence survival (91.0 vs. 101.0 months; *p* = 0.40), although postoperative morbidity was similar between groups. The only subgroup showing a significant interaction favoring HIPEC was non-serous histology (HR 0.29, 95% CI 0.11–0.75). The authors attributed the negative result partly to unusually favorable patient selection (median PFI of 18 months, near-universal complete resection, and 43.5% BRCA-mutation carriers), which may have narrowed the achievable margin of benefit for any added local therapy. Additionally, they noted the absence of preoperative platinum exposure. This finding is directly consistent with the “chemotherapy-priming” hypothesis proposed above to reconcile OVHIPEC-1 and NCT01091636, and is now reinforced by the contrast between CHIPOR (HIPEC after six cycles of platinum-based chemotherapy: positive) and HORSE (HIPEC without prior chemotherapy: negative) in the same platinum-sensitive recurrent setting.

A 2025 meta-analysis pooling six RCTs of HIPEC in epithelial OC (987 patients total, including OVHIPEC-1, the Korean trial, CHIPOR, HORSE, Zivanovic et al., and Cascales-Campos et al.) provides quantitative support for this pattern [30]. The addition of HIPEC significantly improved PFS in patients with newly diagnosed advanced-stage disease who had received neoadjuvant chemotherapy (HR 0.59, 95% CI 0.39–0.88; *p* = 0.01), but conferred no significant PFS benefit in the recurrent setting without prior NACT (HR 1.04, 95% CI 0.72–1.49; *p* = 0.85; and HR 1.22, 95% CI 0.82–1.83 after excluding CHIPOR). HIPEC was associated with a significantly higher risk of acute kidney injury (10.6% overall; OR 4.01, 95% CI 1.62–9.96; *p* = 0.003) and a modestly higher rate of any-grade thrombocytopenia (OR 1.55, 95% CI 1.03–2.33; *p* = 0.03), while rates of infection, sepsis, hemorrhage, and gastrointestinal complications did not differ from surgery alone. Nephroprotection with sodium thiosulfate, incorporated into two of the pooled trials as a protocol amendment, was associated with a reduction in renal adverse events, suggesting a practical mitigation strategy for this specific risk. Taken together, these pooled data indicate that the survival benefit of HIPEC in OC is most consistently observed in patients who have already received platinum-based systemic chemotherapy, whether neoadjuvant or as part of recurrence management, while its added value in chemotherapy-naïve settings, at either primary or secondary surgery, remains unproven and is accompanied by a clinically meaningful increase in renal toxicity. The results of several other studies are presented in Table 1.

### 3.2. Gastric Cancer

Studies are being conducted to evaluate the efficacy of HIPEC combined with CRS in patients with GC. Thus, in a systematic review presented by J. Zhang et al. (2026), data from a meta-analysis of nine clinical studies (*n* = 1021 patients) evaluating the potential role of HIPEC in patients with GC and peritoneal metastases (PM) were reported [37]. In five of the included studies (5/9, 56%), a significant improvement in 3-year survival was demonstrated in the HIPEC group compared with the control group; in three studies (3/9, 33%), an increase in 3-year PFS was observed; in seven studies (7/9, 78%), the incidence of PM was reduced by a factor of 2.4; and in three studies, a lower rate of distant metastases was reported. At the same time, all nine studies indicated that HIPEC was not associated with an increased incidence of adverse events, including neutropenia, liver dysfunction, thrombocytopenia, and renal impairment, while four studies likewise reported no increase in surgical complications, including anastomotic leakage, intestinal obstruction, and surgical site infection, as compared with the control group [37].

In a meta-analysis by Y. Chen et al. (2025), which included 12 studies and 1181 patients, HIPEC was shown to provide statistically significantly higher survival rates at 1, 2, 3, and 5 years compared with the control group [38]. The overall recurrence rate in patients treated with HIPEC was lower than that observed in the comparison group (*p* < 0.0001). In addition, a higher PFS rate was reported following HIPEC (*p* < 0.01) [38]. Similar findings were reported by M. Patel et al. (2023) on the basis of a meta-analysis of six randomized controlled trials and 10 non-randomized studies, encompassing a total of 1700 patients with PC secondary to GC [39]. The authors concluded that HIPEC provides a significant advantage over standard treatment with respect to 3- and 5-year OS, as well as a reduction in the recurrence rate. At the same time, the risk of renal dysfunction was higher in patients receiving HIPEC, whereas differences in the incidence of other complications, including surgical complications, were not statistically significant [39]. In contrast, B. Rau et al. (2024) demonstrated that the use of HIPEC following CRS did not improve OS compared with the control group, but was associated with statistically significant improvements in PFS and metastasis-free survival [40].

Some studies have explored the feasibility of prophylactic HIPEC in patients at high risk of developing peritoneal metastases (PM). Thus, J. Desiderio et al. (2017), having analyzed the results of 11 randomized controlled trials and 21 non-randomized controlled studies involving a total of 2520 patients, showed that, in patients without established PC, 3- and 5-year OS rates were higher in the HIPEC group than in the control group (*p* = 0.01) [41]. In contrast, in patients with established PC, the authors found no difference in 3-year OS between the compared groups; however, median survival was 4 months longer in those who received HIPEC. The HIPEC procedure was associated with a higher risk of systemic complications [41].

A large European phase III clinical trial, GASTRICHIP (NCT01882933), is currently nearing completion. Its results are expected to clarify whether prophylactic HIPEC in patients with locally advanced GC can reduce the incidence of peritoneal dissemination and improve OS [42]. The trial includes two patient groups: those undergoing radical gastrectomy with D1–D2 lymphadenectomy plus oxaliplatin-based HIPEC, and those undergoing radical gastrectomy with D1–D2 lymphadenectomy alone, without HIPEC. The results of this study have not yet been published.

A randomized phase III clinical trial, NCT05871099, is likewise evaluating the efficacy of adding HIPEC to laparoscopic gastrectomy with D2 lymphadenectomy in patients with locally advanced GC for the prevention of peritoneal metastases; however, in this study, two HIPEC sessions and systemic chemotherapy are planned [43]. Actual treatment outcomes have not yet been reported in the available literature. Another phase III study, NCT04597294 (the CHIMERA trial), which is also designed to investigate the prophylactic value of HIPEC, differs in that irinotecan-based HIPEC is administered preoperatively at the stage of diagnostic laparoscopy, following four cycles of neoadjuvant FLOT chemotherapy, whereas after gastrectomy with D2 lymphadenectomy, an additional four cycles of FLOT are given in the adjuvant setting [44]. The results of this trial have not yet been published.

The results of the ongoing and future clinical studies will likely expand our understanding of the potential role of prophylactic HIPEC in patients at high risk of peritoneal metastases (PM). Large multicenter phase III trials with larger patient cohorts, including diverse ethnic populations, are needed to define the role of HIPEC in the treatment of advanced GC. The results of previously published studies should be interpreted with caution, as emphasized by the authors themselves. The principal limitations include the lack of standardization with respect to the chemotherapeutic agents used and their dosages, the duration and temperature of perfusion, the number of treatment sessions, the timing of initiation of postoperative perfusion therapy, as well as the standard extent of surgical intervention. In addition, problems related to blinding and concealment of random allocation remain [37]. Further improvement in HIPEC outcomes will require resolution of the problem of uniform drug distribution within the abdominal cavity, the availability of specialized perfusion equipment, and precise temperature control, while patient access to this method is also determined by insurance coverage of the procedure. At present, there is no consensus as to which patients are most likely to derive the greatest benefit from HIPEC, since specific prognostic markers of the efficacy of this procedure have not yet been definitively established.

According to M. Ikeguchi et al. (1995), patients with positive cytological findings and limited lymph node involvement (fewer than 10 affected nodes) may derive the greatest benefit from HIPEC [45]. Another favorable factor indicating the potential utility of HIPEC in the postoperative setting is the achievement of complete cytoreduction in patients with low-volume disease [46]. In such patients, adjuvant HIPEC has been associated with a doubling of survival and a reduction in the rate of peritoneal recurrence from 73.7% to 11.1%. The authors also demonstrated a survival advantage in patients with peritoneal metastases who underwent aggressive CRS in combination with HIPEC. Similar data emphasizing the critical importance of complete CRS for improving survival in patients with extensive carcinomatosis were reported in study [41].

In contrast, the PERISCOPE I study demonstrated lower efficacy of HIPEC, as reflected by a high recurrence rate (68%) in patients with unfavorable tumor characteristics, including advanced T stage, diffuse histology, and a limited response to systemic chemotherapy [47].

It has been suggested that a Peritoneal Cancer Index (PCI) of 10–13 may be considered a potential criterion for selecting patients for CRS followed by HIPEC. For example, in the study by O. Glehen et al. (2010), patients with a PCI greater than 12 did not achieve 3-year OS, whereas those with a PCI greater than 19 failed to reach even 6-month survival [48]. However, other investigators [49] have questioned the possibility of defining a threshold PCI value for predicting the efficacy of HIPEC. Further research in this area would be valuable for substantiating the incorporation of this method into clinical practice.

Two recent phase III randomized controlled trials have substantially clarified, and tempered, expectations for HIPEC in GC. The GASTRIPEC-I trial randomized 105 patients with synchronous peritoneal metastases from GC to gastrectomy with CRS alone (CRS-A, *n* = 53) or CRS plus HIPEC with cisplatin and mitomycin C (CRS + H, *n* = 52) [40]. Median OS was identical between groups (14.9 months in both arms; hazard ratio 0.72, 95% CI 0.39–1.32; *p* = 0.1647), and the trial found no significant OS benefit from adding HIPEC. However, PFS was significantly longer with HIPEC (7.1 vs. 3.5 months; *p* = 0.047), as was distant metastasis-free survival (10.2 vs. 9.2 months; *p* = 0.029), without any increase in grade ≥ 3 toxicity (43.6% vs. 38.1%; *p* = 0.79). Notably, in the prespecified subgroup of patients who achieved complete cytoreduction (CCR0), HIPEC was associated with a statistically significant OS benefit, again underscoring that completeness of cytoreduction, not HIPEC per se, may be the dominant determinant of outcome.

The subsequent PERISCOPE II trial directly tested this question in a more favorable population: 102 patients with GC and limited peritoneal disease (PCI < 7 or positive peritoneal cytology only) who had not progressed after at least three cycles of systemic therapy were randomized to continued systemic therapy alone or to gastrectomy with CRS and HIPEC (oxaliplatin plus docetaxel) [50]. The trial was stopped prematurely after an unplanned interim analysis for futility. Median OS was numerically similar, if anything favoring systemic therapy alone (16.6 months vs. 15.7 months; hazard ratio 1.10, 95% CI 0.69–1.74; *p* = 0.70), while the surgical arm carried substantially higher toxicity (grade ≥ 3 adverse events, 42% vs. 20%; serious adverse events, 44% vs. 6%) and three treatment-related deaths, all occurring after CRS + HIPEC.

Taken together with the earlier PRODIGE 7 experience in CRC, GASTRIPEC-I and PERISCOPE II indicate that the survival benefit of HIPEC in GC, if it exists at all, is likely confined to a narrow subgroup of patients in whom complete cytoreduction is achievable, and that in patients selected using currently available criteria (low PCI, disease control on systemic therapy), adding CRS and HIPEC to systemic therapy increases toxicity without a demonstrated survival benefit. These findings reinforce the NCCN and ESMO guideline positions discussed in Section 3.5 and argue against routine use of CRS + HIPEC for GC peritoneal metastases outside the context of a clinical trial.

### 3.3. Colorectal Cancer

The results of HIPEC in patients with CRC and PC remain inconsistent. According to F. Quénet et al. (2021), whose study included 265 patients, of whom 133 were assigned to the CRS plus HIPEC group and the remainder to the comparison group, median OS did not differ significantly between the two groups (stratified log-rank test, *p* = 0.99) [51]. The incidence of grade 3 or higher adverse events at 30 days was similar between the groups, whereas at 60 days it was higher in patients who had received HIPEC (*p* = 0.035). The authors concluded that, because the addition of HIPEC to CRS is more frequently associated with late postoperative complications, CRS alone should be considered the principal treatment strategy for peritoneal metastases of CRC [51].

In the study by C. Jian et al. (2023), a meta-analysis of 12 randomized controlled trials was performed, three of which specifically evaluated CRS followed by HIPEC in patients with CRC (617 patients in total) [52]. Combined treatment did not result in a significant improvement in OS in this patient subgroup [52]. In addition, no substantial differences were observed in PFS or RFS. The combination with HIPEC was associated with higher nephrotoxicity, whereas the incidence of other adverse effects was similar in both groups [52]. The authors noted that the patients included in the analyzed studies had not been stratified according to histopathological type and disease stage, which precluded a more detailed interpretation of the data obtained.

A randomized phase III clinical trial (NCT02614534), which included 184 patients, evaluated local control outcomes following HIPEC with mitomycin C administered during CRS followed by systemic therapy, as compared with surgery and systemic therapy alone, in adult patients with locally advanced CRC [53]. Improved local control at 3 years was demonstrated in the HIPEC group (97%) compared with the group treated with surgery alone (87%) (*p* = 0.03). No differences in PFS or OS were observed between the groups; however, among patients with pT4-stage disease, a marked advantage in 3-year PFS was noted following HIPEC therapy (log-rank *p* = 0.003). Treatment-related toxicity was similar in both groups. According to the authors, the combination of HIPEC, complete cytoreduction, and systemic therapy should be considered a potential treatment option for locally advanced CRC.

Attempts have also been made to investigate the potential role of HIPEC in the adjuvant setting in patients with T4N0-2M0 disease or perforated colon cancer. However, according to the available data after 59 months of follow-up [54], the use of HIPEC in combination with adjuvant systemic chemotherapy in these patient groups did not improve 5-year OS or PFS, nor did it reduce the incidence of peritoneal metastases compared with systemic chemotherapy alone, and therefore cannot be recommended as a routine approach.

The PROPHYLOCHIP study evaluated the benefit of repeat surgery combined with HIPEC in patients with CRC at high risk of developing peritoneal metastases. Initial treatment consisted of surgery followed by adjuvant chemotherapy, after which, in the absence of disease progression, patients underwent either observation or further treatment. No significant differences were observed in 3-year peritoneal RFS, OS, or 5-year OS [55]. However, the high rate of postoperative complications (41%) and the potential for oxaliplatin resistance, reflected by the high recurrence rate, argue against the routine use of this approach [56]. N. Tinsley et al. (2025) compared the efficacy of HIPEC with mitomycin versus HIPEC with oxaliplatin in 409 patients with CRC and peritoneal metastases [57]. Median OS did not differ significantly between oxaliplatin- and mitomycin-based HIPEC in previously untreated patients [57]. However, the use of oxaliplatin was associated with a significant improvement in PFS, which supports its preferential evaluation in future studies [57].

The negative result of PRODIGE 7 [51] has been extensively debated, and several methodological limitations of the trial have since been identified that temper its interpretation. First, the trial’s sample size was powered to detect an 18-month increase in median OS (from 30 to 48 months), a substantially larger effect than is typically expected of an oncologic intervention, and no adequately powered trial has been conducted to detect a more realistic, modest benefit. Second, OS may be a suboptimal primary endpoint for a locoregional treatment such as HIPEC: patients in both arms received systemic chemotherapy perioperatively and at relapse, which can dilute any locoregional treatment effect, and peritoneal recurrences in the non-HIPEC arm were themselves treated with salvage CRS + HIPEC, introducing a crossover confounding effect. Notably, 1-year peritoneal RFS favored the HIPEC arm (59% vs. 46.1%), suggesting a locoregional control benefit that the OS endpoint may not have captured. Third, the oxaliplatin-based HIPEC protocol used (460 mg/m^2^ for only 30 min, combined with bolus rather than continuous 5-FU) has been questioned as suboptimal, and preoperative oxaliplatin-based systemic chemotherapy (administered to most trial participants) may itself induce chemoresistance that blunts the efficacy of subsequent intraperitoneal oxaliplatin. Fourth, the trial enrolled patients with PCI up to 25 and included a subset with incomplete cytoreduction (R2 resections) and rectal primaries, whose biology differs from colon cancer; a post-hoc subgroup analysis suggested a significant OS benefit of HIPEC specifically in patients with PCI 11–15 (41.6 vs. 32.7 months, *p* = 0.0209) [51].

These considerations have motivated a new generation of trials designed explicitly to address the flaws attributed to PRODIGE 7. The Spanish GECOP-MMC trial (phase IV, multicenter, ClinicalTrials.gov NCT05250648), currently recruiting across 31 centers, restricts enrollment to colon (not rectal) cancer with PCI ≤ 20 and complete cytoreduction (CCS-0) confirmed intraoperatively, uses high-dose mitomycin-C (35 mg/m^2^, 90 min) rather than oxaliplatin, and, reflecting the concerns raised above, adopts peritoneal RFS at 3 years, rather than OS, as its primary endpoint [58]. Results are not yet available, but the trial’s design itself illustrates the field’s shift toward regimen standardization (following the PSOGI/RENAPE Delphi consensus favoring high-dose mitomycin-C) and toward endpoints that more directly capture the locoregional effect of HIPEC. Pending these results, the evidence for HIPEC in colorectal peritoneal metastases remains genuinely unsettled: PRODIGE 7 does not support routine use of oxaliplatin-based HIPEC after complete CRS, but neither does it exclude a benefit of HIPEC administered with a different agent, longer exposure time, or in a more precisely selected patient subgroup.

### 3.4. Patient Selection Criteria

Beyond PCI, patient selection for intraperitoneal regional therapy differs meaningfully across tumor types. In OC, completeness of cytoreduction (CC-0/CC-1) is the strongest independent prognostic factor, and germline/somatic BRCA and homologous recombination deficiency (HRD) status are increasingly recorded in HIPEC trials given their established role in platinum sensitivity and PARP-inhibitor eligibility. However, whether HRD status specifically predicts benefit from platinum-based HIPEC remains unresolved. Indeed, the post-hoc analysis of OVHIPEC-1 suggested, if anything, a greater HIPEC benefit in BRCA wild-type rather than BRCA-mutated patients, underscoring that biomarker-driven selection is an active area of investigation rather than an established criterion [59]. ECOG performance status of 0–1 and response to prior neoadjuvant chemotherapy serve as pragmatic eligibility thresholds in most active trials. In GC, selection is additionally constrained by tumor biology: intestinal-type histology and the absence of a signet-ring cell component are associated with better outcomes after CRS + HIPEC than poorly cohesive/signet-ring cell carcinoma, which (despite being the predominant histology among patients selected for regional therapy in most trial cohorts) carries a substantially worse prognosis and a lower expected benefit from HIPEC [60]. Peritoneal-predominant (P1) disease without hematogenous spread remains a prerequisite for regional therapy regardless of histology, while HER2 status increasingly informs the choice of concurrent systemic therapy. In CRC, selection is primarily anchored to a PCI ceiling (commonly ≤20 for CRS + HIPEC eligibility) together with the extent of prior systemic chemotherapy, both of which affect operability. RAS, BRAF, and MSI/mismatch-repair status may provide prognostic information and influence the choice of systemic treatment, but they have not been validated as stand-alone criteria for selecting patients for HIPEC. Across all three tumor types, PCI should therefore be interpreted together with surgical resectability, patient fitness (ECOG performance status), prior treatment response, and tumor biology, rather than used as an isolated threshold.

### 3.5. Current Guideline Recommendations

Guideline positions on HIPEC differ markedly by tumor type, reflecting the heterogeneity of the underlying evidence discussed above. For OC, the NCCN Guidelines [61] now include HIPEC as an option for FIGO stage III disease treated with neoadjuvant chemotherapy followed by interval CRS with optimal cytoreduction, based primarily on OVHIPEC-1. Similarly, a 2025 multisocietal consensus using the GRADE framework issued a strong recommendation for HIPEC in this setting. For GC, systemic chemotherapy or best supportive care remains the default NCCN recommendation for peritoneal dissemination. However, since Version 1.2024, the NCCN Guidelines have incorporated a dedicated algorithm for peritoneal-limited disease, allowing CRS with gastrectomy and IPC or HIPEC as an additional option in carefully selected patients, specifically those with a low PCI (≤10) and stable or responding disease after systemic therapy, following multidisciplinary discussion. Notably, PIPAC was subsequently removed from this algorithm in a later revision and is now recommended only within a clinical trial, reflecting the guideline committee’s assessment that supporting evidence in this setting still consists largely of case reports and small series rather than the randomized data available for OC. This selective, low-PCI-restricted endorsement is broadly consistent with the more limited and heterogeneous evidence base discussed in Section 3.2, including the negative GASTRIPEC-I and PERISCOPE II trials. For CRC, the 2023 ESMO Clinical Practice Guidelines recommend complete CRS, but state that IPC (including HIPEC) should be offered only within clinical trials, citing the negative PRODIGE 7 trial. By contrast, the 2022 PSOGI international consensus issued a conditional recommendation for HIPEC in colorectal peritoneal metastases, explicitly advising against short-duration, high-dose oxaliplatin regimens in favor of mitomycin-based protocols. This divergence between European (ESMO) and peritoneal-surface-oncology-specialty (PSOGI) guidance for CRC underscores the ongoing controversy noted in Section 3.3 and reinforces the need for regimen-specific, adequately powered randomized trials rather than a single generic recommendation for or against HIPEC in this disease.

## 4. Pressurized Intraperitoneal Aerosol Chemotherapy

Pressurized intraperitoneal aerosol chemotherapy (PIPAC) is a minimally invasive technique in which chemotherapeutic agents are delivered directly into the abdominal cavity as aerosols under pressure [8]. With PIPAC, the depth of drug penetration reaches 500–600 μm, while tissue concentrations may reach up to 1.70 μmol/g, potentially exceeding the values observed with conventional IPC [62]. At the same time, the duration of drug–tissue contact is increased. Owing to the high local bioavailability achieved with PIPAC, the dose of cytotoxic agents can be reduced, thereby lowering their systemic toxicity [23]. For example, the dose of oxaliplatin administered via PIPAC is approximately 20% of that used during HIPEC [62]. Improved quality of life after PIPAC has been reported in 89% of cases [63]. The key mechanisms underlying PIPAC include [64]:

- optimization of drug dispersion through aerosol delivery;
- enhanced drug penetration due to increased intraperitoneal hydrostatic pressure;
- minimization of blood outflow during drug administration;
- control of the intraperitoneal environment to enable more effective tissue exposure.

**Figure 2:**
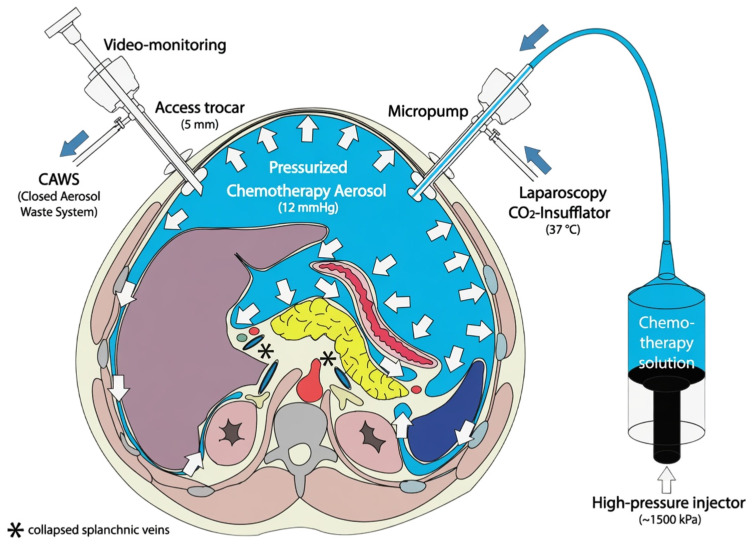
Concept of pressurized intraperitoneal aerosol chemotherapy (PIPAC) treatment in a patient with peritoneal carcinomatosis. The procedure is performed in an operating room equipped with laminar air-flow and is remote-controlled. In the first step, a normothermic capnoperitoneum is established with a pressure of 12 mmHg at body temperature. A chemotherapy solution (about 10% of a normal systemic dose) is nebulized with a micropump into the tightly closed abdominal cavity, and maintained for 30 min. The toxic aerosol is then exhausted through a closed system and released into the external environment. Adapted from [65].

The technical execution and procedural steps of PIPAC are detailed in Figure 2.

Careful patient selection is of critical importance for the performance of PIPAC. Absolute contraindications include [66]:

- bowel or urinary obstruction without the ability to safely and easily bypass or divert, inability to access the abdomen safely, for example because of prior surgery or tumor overgrowth;
- significant abdominal adhesions;
- recent bowel perforation or intra-abdominal sepsis;
- contraindications to laparoscopy due to cardiopulmonary pathology or other conditions precluding surgical intervention. In addition, PIPAC is not recommended in patients without PC, nor in cases where a favorable therapeutic response to systemic therapy has already been achieved [67].

The choice of drug for PIPAC is likewise of major importance. According to the available studies [68], the use of oxaliplatin is more frequently associated with the development of a severe pain, particularly during the first 32 h after PIPAC, as compared with doxorubicin or cisplatin. Severe peritoneal sclerosis, leading to abdominal pain and constipation or bowel obstruction, has occasionally been reported only after oxaliplatin-based PIPAC [69]. Mitomycin [70], taxanes, including nab-paclitaxel [71], irinotecan, and immunotherapeutic agents [67] are currently being considered as potential candidates for evaluation in future studies.

This direction has now been tested clinically. The phase I PIANO trial (NCT03172416) evaluated PIPAC-oxaliplatin (90 mg/m^2^) combined with systemic nivolumab (240 mg every 2 weeks) in 18 patients with GC peritoneal metastases who had progressed on at least first-line systemic therapy [72]. The combination was feasible and safe: grade 3–5 treatment-related toxicity was manageable, and the two grade 5 events observed (bowel perforation and COVID-19-related sepsis) were judged by an independent monitoring committee to be more likely related to disease progression and intercurrent illness than to treatment. A reduction in PCI was observed at the second and third PIPAC procedures (median change −5 and −7, respectively), and peritoneal regression grade 1–2 was achieved in 66.7% and 100% of evaluable patients at these timepoints. Translational analysis of paired peritoneal biopsies demonstrated that the combination altered the tumor microenvironment, with enrichment of immune and stromal infiltration signatures, reduced M2 macrophages, and increased memory CD4+ and CD8+ T-cell populations after treatment, providing early mechanistic support for combining locoregional chemotherapy with checkpoint blockade. However, these immunological and radiological responses did not translate into a survival benefit in this heavily pretreated population (median PFS 1.8 months; median OS 6.0 months), which the authors attributed to the advanced, treatment-refractory nature of the cohort rather than to a lack of biological activity. As the first published trial combining PIPAC with systemic immunotherapy, PIANO establishes feasibility and provides a translational rationale for larger, less heavily pretreated cohorts, but does not yet demonstrate a clinical efficacy signal.

Other complications associated with the PIPAC procedure may include infections, fascial dehiscence, bowel injury during abdominal access, intestinal obstruction, aspiration, and pleural effusion [73], as well as anesthesia-related complications and adverse events associated with the use of cytotoxic agents, including neutropenia, anemia, elevated transaminase levels, nausea, and others [74].

Strict adherence to safety precautions by medical personnel is essential during the performance of PIPAC. It has been demonstrated that contamination with cytotoxic agents may occur on work surfaces, injectors, and trocars, and to a lesser extent in the ambient air [75].

PIPAC was developed as an alternative to HIPEC. It should be noted that both procedures require an operating room and general anesthesia; however, operative time is usually shorter with PIPAC, since it is only rarely accompanied by additional surgical procedures, although in such cases the rate of surgical complications does not increase as compared with PIPAC alone [76]. HIPEC is also associated with a longer hospital stay and a more prolonged postoperative recovery period.

Whereas HIPEC remains the method of choice in cases of optimal cytoreduction (R0/R1) [25], PIPAC is considered preferable in the setting of minimal residual disease or recurrent tumors. Another advantage of PIPAC over HIPEC is that PIPAC can be performed in most patients, including those with an ECOG performance status of 2, whereas HIPEC generally requires a better general condition, namely ECOG 0–1 [77]. In addition, PIPAC can be repeated multiple times, making it possible to assess treatment response and obtain samples of residual tumor tissue. PIPAC may also offer advantages in poorly differentiated tumors because of the improved penetration of aerosolized chemotherapeutic agents [78]. Systemic chemotherapy may be continued without interruption after PIPAC; administration of cytotoxic agents can be resumed as early as 6 days later [79], although in most cases it is restarted after 14 days [67]. It has also been shown that anti-VEGF therapy needs to be discontinued only 2–4 weeks prior to the PIPAC procedure [80]. Taken together, the evidence for HIPEC in GC and CRC remains considerably less consistent than that accumulated for OC. Trials differ substantially in chemotherapeutic agents, perfusion temperature and duration, timing relative to CRS, and completeness of cytoreduction criteria used for enrollment. These methodological variations preclude direct cross-trial comparisons and likely explain part of the discrepancy between positive and negative results summarized above. Studies restricted to patients who achieved complete or near-complete cytoreduction (CC-0/CC-1) consistently show more favorable outcomes than unselected cohorts, indicating that completeness of cytoreduction is a major prognostic factor and a potential effect modifier that should be accounted for when interpreting the incremental benefit of HIPEC. Until future trials adopt harmonized perfusion protocols and prospective stratification by completeness of cytoreduction, the benefit attributable specifically to HIPEC in GC and CRC will remain difficult to establish with confidence. A comparative overview of these methods is presented in Table 2.

A limitation of this method remains the heterogeneous distribution of drugs across the peritoneal surface and their uneven penetration into tissue [82]. Although distribution is more uniform than with IPC, up to 97.5% of the aerosolized fluid may deposit on the tissue surface directly beneath the nebulizer used for cytostatic drug delivery. To improve treatment outcomes, rotation of the nebulizer during aerosolization has been proposed, thereby changing the direction of drug delivery into the abdominal cavity [83]. Another obstacle to the use of PIPAC is the lack of laparoscopic access to the abdominal cavity after previous surgery; the highest risk is observed in patients who have previously undergone CRS and HIPEC [81]. It has been shown that access to the abdominal cavity is not feasible in up to 22% of cases [77].

At present, because of the lack of high-quality evidence, PIPAC is not considered a standard treatment for PC. However, available studies indicate that PIPAC is safe, may stabilize or improve patients’ quality of life, and can provide an objectively measurable reduction in disease burden [84]. Notably, even in phase I clinical trials, no signs of renal or hepatic toxicity were observed with PIPAC, and repeated procedures showed no evidence of cumulative organ toxicity [85].

In the clinical trial NCT03210298, 142 PIPAC procedures using cisplatin, doxorubicin, and/or oxaliplatin were performed in 71 patients with various solid malignancies [81] who had previously received anticancer therapy. The study showed that, in patients with peritoneal metastases, PIPAC may serve as a salvage treatment, improving survival outcomes and reducing ascites beginning after the first treatment cycle. Regression or stabilization of tumor lesions was achieved in 67% of cases (with response assessed in 36 patients), and complete histological tumor regression was observed in 26% of cases. Median survival from the first PIPAC procedure was 6.8 months in patients with ovarian and GC, 11.8 months in those with hepatobiliary-pancreatic tumors, and was not reached in patients with CRC, pseudomyxoma peritonei, or mesothelioma. The rate of intraoperative complications was 2.8%, while postoperative complications occurred in 4.9% of cases; one patient died on postoperative day 12 as a result of cardiopulmonary decompensation. The authors emphasized that tumor regression in peritoneal lymph nodes was achieved despite administration of a cytostatic dose reduced by one order of magnitude, i.e., tenfold lower than the systemic dose, which contributed to good tolerability of the procedure and treatment safety. The strengths of this study include the opportunity to analyze PIPAC outcomes in patients with a variety of tumor types and clinical conditions, including, for example, the post-transplant setting; the first evidence supporting the prognostic significance of the Peritoneal Regression Grading Score (PRGS); and the possibility for participating centers to receive annual reports comparing their own data with overall study benchmarks, thereby contributing to improvement in the quality of care. The limitations of the study include the absence of a control group, heterogeneity of the treatments received by the patients, the use of surrogate markers to assess survival outcomes, and the fact that the principal conclusions were based on a relatively small patient cohort. Owing to the lack of randomization and the absence of a control group, the study design does not permit the conclusion that PIPAC is superior to standard therapy.

The efficacy of PIPAC with cisplatin and doxorubicin was evaluated in 192 patients with platinum-sensitive and platinum-resistant recurrent or progressive OC who had previously received anticancer therapy [86]. OS was 22 months in the PS group and 11 months in the platinum-resistant group (*p* = 0.01). In patients with platinum-sensitive disease, survival was higher when at least 3 cycles of PIPAC could be administered; the same pattern was also observed in the platinum-resistant subgroup. Median PFS was 12 versus 7 months (platinum-sensitive vs. PR, *p* = 0.033). At the same time, PFS did not differ significantly between platinum-sensitive and platinum-resistant patients when 3 or more cycles of PIPAC were administered (median, 16 vs. 13 months, *p* = 0.47). Independent adverse prognostic factors associated with reduced OS and PFS in both subgroups were the presence of ascites and positive cytological findings at the first PIPAC procedure. The authors reported a low incidence of severe complications (4–8%) and mortality (0–3%) in this study and proposed that PIPAC be considered a safe palliative treatment option for advanced OC. The strengths of the study include the inclusion of a large international cohort of patients (234 at baseline) from 18 centers, which increased the statistical power and generalizability of the findings; confirmation of the safety and feasibility of PIPAC in severely ill patients; identification of factors influencing overall and PFS; and the objective assessment of treatment response on the basis of a reduction in the PCI. The limitations of the study include its retrospective design, which increases the risk of systematic bias due to the absence of a control group and a standardized treatment protocol, the heterogeneity of the compared groups, the use of systemic chemotherapy, which makes it difficult to determine the specific contribution of PIPAC to improved survival, and the reduction in the number of patients included in the final analysis after exclusion of early relapses. Owing to its retrospective design and the lack of a control group, this study likewise does not allow a definitive conclusion that PIPAC is more effective than current treatment approaches. The hypothesis generated by the investigators requires confirmation in further high-quality studies.

In study [87], PIPAC combined with intravenous chemotherapy was used to treat PC in patients with various malignancies for whom radical surgery was not feasible. The study included 25 patients, and 58 PIPAC procedures were performed. Tumor regression was achieved in 56% of cases, and in 28% of patients, CRS was performed after two PIPAC procedures. The authors reported a reduction in ascites volume after the first PIPAC procedure (*p* = 0.001), as well as decreases in the PC index (*p* = 0.016) and the peritoneal regression index (*p* = 0.047) during repeated treatment courses. Median OS was 9.6 months; moreover, in cases with a response to treatment, improvements in median OS (*p* < 0.001), PFS (*p* < 0.001), and quality of life (*p* = 0.003) were observed. Unlike retrospective cohort studies, this investigation was designed as a prospective clinical trial, thereby reducing the risk of systematic bias and enhancing the strength of the evidence. A high response rate to therapy was documented during the course of the study, including clinically meaningful control of ascites, which directly affects patients’ quality of life. The prognostic value of PRGS was also demonstrated, with patients showing a pathological response (PRGS 1–2) exhibiting better clinical outcomes than non-responders (PRGS 3–4). PIPAC was shown to have a favorable safety profile, with severe surgical complications (Clavien–Dindo ≥ 3b) observed in only 1.7% of cases, CTCAE grade 3 toxicity in 3.4%, no deterioration in quality of life according to patient-reported outcomes, and no treatment-related mortality.

However, the conclusion regarding the efficacy of PIPAC is based on comparisons with historical data and intragroup analyses, which does not allow superiority over existing standards of care to be established. The small sample size and tumor heterogeneity further complicate interpretation of the findings and preclude drawing conclusions regarding the efficacy of PIPAC in any specific tumor type. The high screen-failure rate prior to the first procedure meant that the analysis was limited to patients with relatively “favorable” abdominal anatomy, which may have led to an overestimation of the feasibility of the method. Additional limitations of the study include its single-center design, the absence of comparison with CRS plus HIPEC, and the limited scope of survival assessment. Although the study demonstrates the safety and potential efficacy of PIPAC, it does not answer the question of whether PIPAC is superior to standard therapy or to CRS combined with HIPEC. Further studies are required to determine whether this approach offers a survival advantage.

The potential use of PIPAC in the neoadjuvant setting is also being explored. In the study by F. Tidadini et al. (2023), 100 PIPAC procedures with oxaliplatin or doxorubicin–cisplatin were performed in 49 patients with PC arising from various tumor types, with a median of 2 procedures (range, 1–3) per patient; 28 patients (57.1%) underwent more than one PIPAC procedure [88]. As a result, the PCI decreased in 37% of cases, remained stable in 29.6%, and increased in 33.4% [88]. Subsequently, four patients (8.3%) underwent radical R0 resection. In the study by M. Alyami et al. (2021), 437 PIPAC procedures were performed in 146 patients with gastric, peritoneal mesothelioma, ovarian, colorectal, and small-bowel tumors; among them, 26 patients (17.8%) underwent a total of 76 PIPAC procedures [89]. All patients received systemic chemotherapy between PIPAC sessions. Subsequent complete cytoreduction and HIPEC became feasible in 14.4% of cases (21 of the 26 patients initially considered for this approach), whereas the remaining five patients were deemed unresectable at diagnostic laparotomy. Among the patients who underwent cytoreduction and HIPEC, 66.7% remained recurrence-free after 7 months of follow-up [89]. The authors concluded that complete cytoreduction and HIPEC may be feasible in highly selected patients who initially present with unresectable metastases following repeated palliative PIPAC procedures. According to the study by R. Sindayigaya et al., CRS and HIPEC could be performed after PIPAC in 10 patients (7%), with a median OS of 15 months (range, 4–27 months) [90]. According to these investigators, receipt of three or more PIPAC procedures constituted an independent prognostic factor associated with improved OS (hazard ratio, 0.36; *p* < 0.0001). Other authors have likewise reported a favorable effect of an increased number of PIPAC cycles on patient survival [91]. This may be facilitated by administering PIPAC before the onset of chemoresistance; the use of PIPAC at earlier stages of treatment may allow its combination with systemic chemotherapy and potentially optimize both local and systemic tumor control [92].

The potential role of prophylactic PIPAC has also been investigated. For example, M. Graversen et al. (2023) applied PIPAC after laparoscopic D2 gastrectomy in 21 patients with high-risk gastric tumors, defined as a poorly cohesive subtype with predominance of signet-ring cells, clinical stage ≥ T3 and/or ≥N2, or positive peritoneal cytology [93]. The authors reported no cases of positive cytology after PIPAC, which, in their opinion, indicates a substantial antitumor effect of the procedure. By contrast, after gastrectomy, the rate of positive cytological findings may reach as high as 60%. Nevertheless, high-quality studies with larger patient cohorts, a more homogeneous background treatment profile, clearly defined timing of treatment initiation, a standardized number of treatment cycles, and appropriate control groups are required in order to determine whether prophylactic PIPAC offers a genuine clinical advantage.

The results of several studies involving PIPAC combined with chemotherapy in patients with solid tumors are presented in Table 3. Thus, PIPAC combined with chemotherapy is a safe procedure in pretreated patients with solid malignancies and may represent an effective maintenance treatment option in patients with primary advanced inoperable OC. Further optimization of drug combinations used during PIPAC is needed, as well as identification of optimal patient selection criteria to determine those most likely to benefit from this method and the identification for molecular targets that may prove useful for predicting treatment outcomes. Clinical trials evaluating the efficacy of PIPAC in combination with systemic chemotherapy are warranted.

A second ongoing randomized phase III trial, PIPAC VER-One (EudraCT 2021-000830-33; NCT05303714), is evaluating PIPAC specifically in oligometastatic GC with synchronous, low-burden peritoneal disease (PCI ≤ 6 and/or positive peritoneal cytology) [105]. Patients are randomized to standard systemic chemotherapy (FOLFOX) alone versus the same chemotherapy combined with PIPAC (cisplatin and doxorubicin) every two cycles, with radical-intent gastrectomy and CRS plus HIPEC offered to responders in either arm. The primary endpoint is secondary resectability rate. This primary endpoint reflects the trial’s core hypothesis (informed by subgroup findings from the CYTO-CHIP study suggesting that patients with low PCI and non-poorly-cohesive histology benefit most from CRS plus HIPEC) that adding PIPAC to systemic chemotherapy in this favorable subgroup may convert a larger proportion of patients to radical resectability, rather than aiming to demonstrate a direct survival benefit of PIPAC itself. Together, PICCOS and PIPAC VER-One represent the first generation of adequately powered randomized trials to test PIPAC’s role as an adjunct to, rather than a substitute for, systemic therapy and definitive surgery. Their results should substantially clarify the currently uncertain place of PIPAC in treatment algorithms for gastric and colorectal peritoneal metastases.

Across the studies reviewed, PIPAC is consistently associated with a favorable safety profile and objective evidence of drug penetration into tumor tissue, but comparative evidence of a survival benefit over systemic chemotherapy alone remains limited to small, heterogeneous, mostly single-arm or retrospective series. Reported response rates and changes in PCI vary widely depending on the agent used (cisplatin/doxorubicin, oxaliplatin, or nab-paclitaxel), number of cycles administered, and baseline tumor burden, with few studies reporting the survival endpoints needed for cross-comparison with HIPEC. Consequently, PIPAC should currently be regarded as an investigational locoregional approach used predominantly in the palliative setting and, in selected studies, as a potential bridging strategy, rather than an established alternative to HIPEC. Randomized comparisons directly addressing this question are still lacking.

## 5. Electrostatic Precipitation Pressurized Intraperitoneal Aerosol Chemotherapy

**Figure 3:**
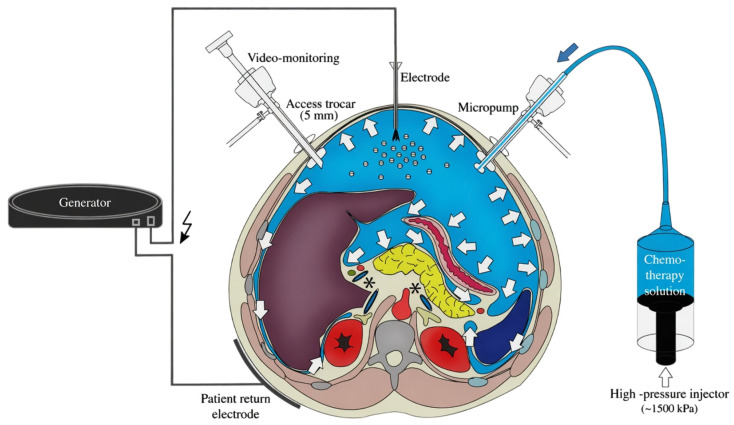
Concept of electrostatic precipitation pressurized intraperitoneal aerosol chemotherapy (ePIPAC) treatment in a patient with peritoneal carcinomatosis. Technical setting for ePIPAC, including high-pressure injector containing therapeutic solution micropump generating pressurized intraperitoneal aerosol, brush electrode for electrostatic loading of therapeutic aerosol, and return electrode. * Collapsed splanchnic veins. Adapted from [106].

According to study [106], the outcomes of PIPAC may be improved by the use of an electrostatic field, since this approach increases cellular drug uptake and improves spatial drug distribution. The potential role of electrostatic precipitation pressurized intraperitoneal aerosol chemotherapy (ePIPAC) in the treatment of patients with solid tumors continues to be investigated. In trial NCT03246321, 135 ePIPAC procedures were performed in 48 patients with various malignancies, with concomitant chemotherapy administered in 58.3% of cases. Treatment response or disease stabilization was achieved in nearly half of the patients, with moderate treatment-related toxicity [107]. The technical configuration and intraoperative application of ePIPAC are presented in Figure 3.

According to A. Taibi et al. (2021), 69 patients underwent 147 ePIPAC procedures with oxaliplatin or a cisplatin + doxorubicin combination for CRC, GC, and OC; concomitant systemic chemotherapy was used in 76% of cases [108]. A complete (PRGS1) or major (PRGS2) histological response was observed in 38.5% and 53.8% of cases, respectively, with satisfactory treatment tolerability [108]. A phase I trial, NCT05395910 [109], is also being conducted to determine the safety and tolerability profile of PIPAC/ePIPAC with paclitaxel in previously treated patients with PC.

Further clinical trials will likely clarify the clinical significance of ePIPAC in the multimodal treatment of malignant neoplasms and help define patient selection criteria for the use of PIPAC versus ePIPAC in patients with PC.

## 6. Photodynamic and Photothermal Therapy

**Figure 4:**
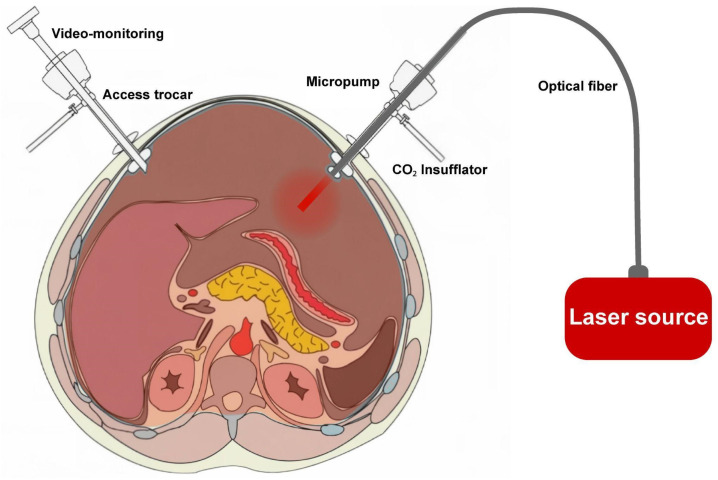
Schematic representation of the laparoscopic setup used for intraperitoneal photodynamic and photothermal therapy of peritoneal carcinomatosis. Light from an external laser source is delivered into the abdominal cavity via an optical fiber introduced through a dedicated access trocar equipped with a micropump for photosensitizer or photothermal agent administration. Pneumoperitoneum is maintained using a CO_2_ insufflator, and the procedure is performed under video-monitoring guidance through a separate access trocar. The same laparoscopic platform can be used for both photodynamic therapy (with a photosensitizing agent) and photothermal therapy (with a photothermal conversion agent), depending on the agent administered and the irradiation wavelength selected.

The advantages of photodynamic therapy (PDT) include low systemic toxicity, except for cutaneous photosensitivity, rapid tissue healing [110], the possibility of laparoscopic guidance, and minimal invasiveness. The therapeutic basis of PDT lies in the ability of photosensitizers (PSs), activated by light of a specific wavelength, to generate high levels of reactive oxygen species (ROS) in tumor tissues through type I or type II photochemical reactions, with the formation of superoxide anion or singlet oxygen, respectively [111] (Figure 4).

ROS exert marked cytotoxic effects. They induce oxidative damage to proteins, lipids, and nucleic acids, leading to malignant cell death through apoptosis or necrosis and destruction of stromal tumor components [112]. Damage to the tumor vasculature through ROS-mediated injury to endothelial cells results in vascular collapse, thrombosis, vascular stasis, and hypoxia [113,114]. In addition, PDT enhances the antitumor immune response, thereby combining tumor ablation with an immunotherapeutic effect [115]. It has been noted that disease recurrence rarely develops in areas treated with PDT [116].

This method is well suited for the treatment of PC because PC lesions are usually small and superficial [117], and the selective uptake of the PS by malignant cells, targeted irradiation, and the limited depth of light penetration into tissues, with the therapeutic effect confined to the irradiated area and a few millimeters below it, may selectively destroy tumor tissue while minimizing damage to normal tissues [110]. The main technical challenge of PDT is the delivery of an adequate therapeutic light dose to all lesions within the complex anatomy of the abdominal cavity.

These technical advantages are counterbalanced by substantial limitations. Light penetration in tissue is restricted to only a few millimeters (typically 3–5 mm depending on wavelength and tissue optical properties) [118]. Consequently, PDT and PTT are inherently unsuited to bulky or deeply infiltrating disease, remaining effective mainly against microscopic or thin superficial deposits. Tumor hypoxia, which is common in poorly vascularized peritoneal implants and can be exacerbated by PDT-induced vascular collapse, directly limits the type II photochemical reaction and reduces ROS generation, creating a self-limiting cycle that attenuates efficacy in larger or centrally hypoxic lesions.

The complex, folded geometry of the peritoneal cavity (with the bowel, mesentery, and diaphragmatic surfaces at variable distances and angles from the light source) makes homogeneous light delivery to all peritoneal surfaces technically difficult, resulting in underdosed regions prone to recurrence and overdosed regions at risk of thermal or photochemical injury to adjacent viscera. Real-time dosimetry (e.g., fluence monitoring) has been used in only a minority of studies, and the marked variability in PS choice, dosing, and drug-light intervals further precludes standardization of treatment protocols across centers. Finally, PDT and PTT require dedicated laser sources, fiber-optic delivery systems, and specialized laparoscopic expertise, which restricts their availability to tertiary centers and complicates the design of multicenter trials, likely contributing to the absence of phase III trials in this setting to date.

Clinical experience with PDT in the treatment of PC remains limited. Nevertheless, the available literature includes the results of phase I–II studies in patients with PC caused by OC, gastrointestinal malignancies, primary peritoneal carcinoma, sarcoma, and recurrent colorectal and gynecologic malignancies. No phase III studies in oncology patients were identified in the available literature. The results of these studies are presented in Table 4. Several different first-generation PSs have been used across studies, including Photofrin (porfimer sodium), haematoporphyrin derivative (HpD), meta-tetrahydroxyphenylchlorin (m-THPC), and chlorin e6 derivatives, administered at doses ranging from 0.15 to 5 mg/kg and at intervals of 2 to 96 h before irradiation, which further underscores the lack of standardization discussed above.

Unlike the studies summarized above, which applied PDT to macroscopic or grossly resected tumors, J.T. Allardice et al. (1994) investigated PDT as a truly adjuvant, prophylactic procedure, irradiating the macroscopically normal tumor bed after colorectal resection to eradicate microscopic residual disease at the resection margin, rather than treating visible tumor [119]. This rationale parallels the prophylactic use of PIPAC discussed above, in which treatment targets subclinical residual disease rather than established macroscopic lesions. Although not designed to formally assess local recurrence, the study reported no local recurrence in 8 of 9 patients with histologically confirmed microscopic margin involvement, a notably favorable outcome compared with historical local recurrence rates for this margin status, and laid the methodological groundwork (light dosimetry, delivery systems, and safety data) for a subsequent phase III trial.

The first head-to-head comparison of intraoperative PDT with HIPEC was reported by D.V. Sidorov et al. (2020) in 57 patients with disseminated mucinous peritoneal pseudomyxoma of appendiceal origin undergoing CRS, of whom 32 received intraoperative PDT (using Photogem or Photosens as the PS) and 25 received HIPEC with cisplatin [123]. Notably, the PDT group had a significantly higher baseline PCI and a substantially lower rate of optimal cytoreduction than the HIPEC group, reflecting non-randomized allocation based on disease extent and resectability rather than a balanced comparison. Despite this less favorable baseline profile, the PDT group showed a significantly lower postoperative complication rate (9.4% vs. 24%) and no perioperative mortality (vs. 12% in the HIPEC group), although 5-year OS was lower (65.2% vs. 86.6%). The authors concluded that, given the markedly more advanced disease burden in the PDT group, intraoperative PDT represents a comparatively safe and effective option in patients for whom optimal cytoreduction is not achievable and HIPEC may therefore be contraindicated or carry unacceptable risk. These findings should be interpreted with caution given the non-randomized design and the imbalance in baseline disease extent between groups, but they provide the first direct clinical comparison between PDT and HIPEC as intraoperative adjuncts to CRS and highlight PDT as a potential option in patients with more extensive peritoneal disease who are poor candidates for HIPEC.

In an effort to reduce PDT-related toxicity, the concept of metronomic photodynamic therapy (mPDT) has been proposed in preclinical studies. In mPDT, the PS and light are delivered continuously at a low rate over extended periods in order to increase the selectivity of tumor cell eradication through apoptosis [125]. A similar strategy may also be applicable in patients with PC, provided that the PS is administered intraperitoneally over an extended period and implantable devices are used to ensure prolonged, uniform, low-dose irradiation of tumor lesions throughout the abdominal cavity. Potential limitations of mPDT may include aggressive tumor growth and, possibly, the risk of an insufficient antitumor immune response [126].

In a conference report, A. Privalov et al. (2017) [127] compared Photolon-mediated PDT (630–680 nm) with HIPEC after CRS in patients with OC-related PC (*n* = 25 and *n* = 21, respectively), with both groups receiving identical neoadjuvant and adjuvant chemotherapy regimens. Recurrence rates were similar between groups (44% vs. 38%), while median time to progression (11.6 vs. 13.5 months) and median OS (18.4 vs. 24.1 months) were somewhat higher with HIPEC, leading the authors to conclude that PDT offered no significant survival advantage over HIPEC for peritoneal metastases.

It is clear that further studies aimed at optimizing the efficacy and safety profile of intraperitoneal PDT should be homogenized with respect to the type of primary cancer, prior treatment, and reported outcomes [128]. It is also necessary to standardize the choice of PS, the interval between PS administration and irradiation, the procedure duration, the irradiation wavelength, the light dose and dose rate, and to optimize light delivery to lesions within the abdominal cavity [110]. In this regard, the development of reliable real-time dosimetry, enabling intraoperative monitoring of PS fluorescence, tissue oxygenation, and the delivered light dose, represents a critical step toward personalizing and standardizing PDT protocols [129,130]. Since clinical studies have so far been conducted using first-generation PSs, it remains to be determined whether second- and third-generation PSs possess sufficient specificity and efficacy for the treatment of PC. Similar translational challenges, including the need to standardize PS selection, irradiation parameters, dosimetry, and clinical outcome assessment, have also been emphasized in recent reviews of PDT for precancerous and malignant gynecological diseases [131]. Addressing these issues may expand the potential role of PDT in patients with PC.

A novel direction of investigation is represented by the Japanese multicenter phase II study jRCT2051220176, which is designed to test the hypothesis of synergy between PDT and immunotherapy, as well as the possibility of “reprogramming” the tumor microenvironment [132]. The study is intended to evaluate a combination of immunotherapy (nivolumab ± ipilimumab or chemotherapy) with Talaporfin sodium-mediated PDT in patients with advanced or recurrent esophageal and GC who are not candidates for radical treatment. The primary endpoint is the overall response rate according to treatment modality. Inclusion of patients whose tumors have progressed during nivolumab monotherapy may provide insight into the efficacy of this combination in the setting of refractory disease. The protocol includes strict exclusion criteria, such as aortic invasion, fistulas, and photosensitization, thereby minimizing the risk of severe complications, including bleeding or perforation [132]. However, the absence of a control group, heterogeneity of the patient cohorts with respect to tumor histology, and the use of different chemotherapy regimens are likely to complicate interpretation of the results and will probably necessitate subsequent phase III clinical trials before this combination can be incorporated into routine clinical practice.

While PDT relies on photochemical generation of ROS to induce tumor cell death, an alternative light-based locoregional strategy—photothermal therapy (PTT)—exploits the conversion of light energy into heat. Both modalities share several practical advantages for the treatment of PC, including compatibility with laparoscopic or minimally invasive delivery, selective activation confined to the irradiated tumor-bearing peritoneal surfaces, and the potential to be combined with immunotherapy or other locoregional treatments discussed above. However, the underlying mechanism of tumor destruction, and consequently the agents and irradiation parameters employed, differ substantially between the two approaches.

PTT is a minimally invasive technique that employs materials with high photothermal conversion efficiency, which can accumulate in tumor tissue through targeted recognition strategies. Upon exposure to external light sources, such as near-infrared light, these materials convert light energy into heat, thereby inducing local hyperthermia and triggering necrosis or apoptosis of malignant cells while minimizing toxicity to healthy tissues [133]. At the same time, a pronounced immune response is observed, contributing to inhibition of both primary and metastatic tumor growth [134]. PDT may increase the sensitivity of tumor cells to PTT by modulating the tumor microenvironment, whereas the heat generated during PTT may enhance blood flow, improve oxygen supply, and potentiate the therapeutic effect of PDT [135]. Under experimental in vitro/in vivo conditions, nanoparticle-based PTT has been shown to effectively control tumor growth and metastasis in OC [136], breast cancer [137], cervical cancer [138], and other malignancies. Studies are currently investigating the combination of PTT using nanomaterials with PD-1/PD-L1 blockade [139], as well as the integration of PDT, PTT, and immunotherapy [135]. However, the promising results obtained in preclinical studies using animal models cannot always be reproduced in the clinical setting. A number of methodological and technical issues related to PTT remain unresolved [140], and addressing them appears essential before clinical trials are initiated.

## 7. Promising Strategies for Improving the Efficacy of Peritoneal Carcinomatosis Therapy

Several promising therapeutic strategies for PC should be highlighted, the further development of which may improve treatment outcomes in oncology patients. Innovative drug delivery systems in the form of micro- and nanomedicinal agents for intraperitoneal administration represent one such approach [8]. Such systems may prolong the residence time of therapeutic agents within the abdominal cavity and enhance targeted drug delivery to tumor tissue, thereby providing a more favorable balance between efficacy and toxicity. Under experimental conditions, polymeric and lipid-based nanocarriers with specific surface modifications, as well as microparticles and hydrogel-based nanocomposites, are being investigated. An example of such compounds is FRRG-doxorubicin nanoparticles (PNPs), which are specific for cathepsin B; in these nanoparticles, doxorubicin is present as a prodrug and is released through sequential enzymatic degradation by cathepsin B and intracellular proteases [141]. PNPs demonstrated cytotoxicity in OC cell lines (SKOV-3 and HeyA8) and colon cancer cell lines (MC38 and CT26) with cathepsin B overexpression, as well as in in vivo models (BALB/c nu/nu and BALB/c mice bearing human ovarian tumor xenografts and patient-derived xenografts). Following intraperitoneal administration, PNPs effectively inhibited tumor growth with minimal adverse effects owing to their high specificity for malignant cells and a favorable in vivo pharmacokinetic/pharmacodynamic profile.

An example of intraperitoneal drug delivery using a lipid nanocarrier is NanoTalazoparib (NanoTZ), which was tested in a murine model (C57BL/6 mice) of late-stage OC with PC [142]. The formulation demonstrated efficacy in reducing the volume of intra-abdominal tumor deposits compared with both untreated controls and free talazoparib, as well as in decreasing the number of disseminated tumor implants on the peritoneal surface. NanoTZ also contributed to a reduction in ascites volume, while exhibiting longer retention within the abdominal cavity than free talazoparib. In addition, NanoTZ did not induce marked weight loss in mice, was not associated with significant alterations in blood parameters, and showed no evidence of pronounced organ toxicity [142]. Nanogels have likewise attracted considerable attention as delivery systems because of their high drug-loading capacity, biocompatibility, stability, and sensitivity to microenvironmental conditions. One such example is an alginate-based cisplatin nanogel encapsulated within an in situ cross-linkable alginate-based hydrogel matrix [143], which was evaluated in a murine model of PC using C57BL/6 mice and the ID8-KRAS cell line harboring the KRAS G12V oncogenic mutation. Prolonged retention of the nanogel within the abdominal cavity, together with sustained controlled release of cisplatin, enabled prolonged antitumor activity, as confirmed under in vivo experimental conditions. The developed formulation exhibited significantly lower toxicity than the free drug.

However, the enhanced permeability and retention (EPR) effect cannot always be exploited, since tumor lesions vary in size and localization and often exhibit poor vascularization and perfusion [144]. To improve treatment efficacy, active targeting is being employed through the addition of various molecules or even whole cells to the nanoparticle surface in order to interact with specific ligands or targets overexpressed by the tumor. An example of this approach is a unique “carrier cell + nanoparticle” delivery system—the use of cisplatin-loaded silica nanoparticles conjugated with tumor-tropic neural stem cells (NSCs), which provides higher cisplatin concentrations in ovarian tumors [145]. In addition, compounds targeting the tumor microenvironment are currently under development [146].

Clinical trial NCT00825201 demonstrated the feasibility of intraperitoneal administration of nab-paclitaxel (albumin-bound paclitaxel nanoparticles) for the treatment of patients with PC [147]. The drug concentration in the abdominal cavity was approximately 150-fold higher than that in plasma, owing to the fact that nanoparticles measuring approximately 130 nm cannot be rapidly absorbed into the peritoneal blood capillaries and are cleared predominantly via the lymphatic system, which markedly slows their elimination from the peritoneal cavity. In addition, due to its albumin-based formulation, the drug maintains therapeutic concentrations in the systemic circulation, thereby enabling antitumor activity against micrometastases located beyond the abdominal cavity. Use of the drug was associated with a more favorable safety profile than conventional paclitaxel. Overall, the study demonstrated promising clinical activity in patients with advanced malignant neoplasms predominantly confined to the abdominal cavity.

Gene delivery systems aimed at suppressing the expression of specific genes in tumor or immune cells represent another avenue of investigation, with the goal of promoting direct or immune-mediated destruction of malignant cells [8]. Such systems include viral and nonviral vectors, as well as nonviral nanoparticles, particularly lipid and polymeric nanoparticles [148]. For example, Singh et al. (2021) used lipid nanoparticles (LNPs) encapsulating a combination of small interfering RNAs targeting polo-like kinase 1 and the C subunit of eukaryotic translation initiation factor 3, additionally modified with hyaluronan to target CD44-expressing cells [149]. Treatment of 2D and 3D cultured OC cell cultures with these LNPs led to the death of 85% of tumor cells and marked suppression of target genes, while intraperitoneal administration of LNPs in a model of advanced orthotopic OC increased animal survival. Gene therapy may also be directed against components of the tumor microenvironment, for example myeloid cells [150].

Novel combinations of PIPAC and ePIPAC with other advanced peritoneal delivery methods are also being explored, including PIPAC combined with mRNA lipoplexes [151] and PIPAC/ePIPAC with polylactic-co-glycolic acid nanoparticles loaded with curcumin [152]. The combination of PIPAC and nab-paclitaxel has demonstrated a favorable pharmacokinetic profile and antitumor activity [153].

Innovative photoimmunoconjugates and nanocarriers loaded with PSs represent a further direction of development. Crystalline aluminum phthalocyanine nanoparticles are an example of a theranostic nanoscale PS platform combining fluorescence-based visualization with light-induced cytotoxicity [154]. An example of such a compound is the IR700 antibody conjugate (IRDye700DX), a near-infrared PS that selectively induces rapid tumor cell death in malignant cells expressing a specific antigen, such as EGFR or HER2 [155].

Light delivery innovations, including implantable light panels and laser beams with a diffusing tip, as well as the use of PDT in combination with high-energy ionizing radiation, for example radiotherapy, are also under active investigation [156]. For the treatment of solid tumors, combinations of PDT with chemotherapy, chemoimmunotherapy, and immune checkpoint inhibitors are being evaluated [111]. Under experimental conditions, it has been shown that local in situ PDT followed by adoptive transplantation of immature dendritic cells may represent a promising therapeutic strategy for the treatment of peritoneal tumors [157].

Despite these encouraging preclinical results, translation of nanomedicine-based delivery systems into clinical practice for PC remains limited by several unresolved challenges. Most formulations described above have been evaluated only in murine xenograft or syngeneic models. Biodistribution, clearance kinetics, and toxicity profiles established in these preclinical models do not reliably predict behavior in the human peritoneal cavity, where tumor burden, adhesions, and prior surgery substantially alter drug distribution. Furthermore, GMP-compliant, reproducible large-scale manufacturing of nanoparticles with consistent size, surface chemistry, and drug loading remains technically demanding and costly. This limitation has restricted most agents to phase I feasibility studies rather than randomized comparative trials. Active-targeting strategies that rely on cell-surface antigen expression (such as HER2 or cathepsin B substrates) also face intratumoral and intertumoral heterogeneity, which may limit efficacy in unselected patient populations. For immune checkpoint inhibitors combined with intraperitoneal or locoregional therapy, additional obstacles include an immunosuppressive peritoneal tumor microenvironment, a paucity of validated predictive biomarkers, and the risk of immune-related adverse events. Addressing these barriers will require standardized, scalable formulations and early-phase clinical trials specifically designed to characterize human intraperitoneal pharmacokinetics before these strategies can be integrated into routine clinical practice.

Beyond drug-delivery innovations, patient selection for locoregional therapy is beginning to incorporate molecular and liquid-biopsy biomarkers rather than anatomical criteria alone. Circulating and peritoneal tumor DNA (ctDNA and ptDNA) have been shown to predict response to HIPEC and serve as independent predictors of PFS in patients with peritoneal metastases. Consequently, these assays are increasingly proposed as non-invasive tools for treatment monitoring and recurrence detection in colorectal peritoneal metastases [158,159]. When combined with germline or somatic BRCA and HRD status in OC, as well as MSI and HER2 status in GC and CRC, such biomarkers may enable dynamic, rather than purely anatomical, patient stratification for regional therapy.

Combinations of locoregional therapy with immune checkpoint inhibitors are also entering early clinical testing. The phase I PIANO trial combined PIPAC-oxaliplatin with systemic nivolumab in GC peritoneal metastases, demonstrating feasibility alongside early translational evidence of immune activation within the peritoneal compartment [72]. Similar strategies combining HIPEC with checkpoint inhibitors and systemic chemotherapy are currently being evaluated in prospective phase II trials for GC peritoneal metastases [160], building on the IPC plus PD-1 blockade data discussed in Section 2. These approaches will need to overcome both the immunosuppressive peritoneal microenvironment and the absence of validated predictive biomarkers noted above.

Finally, artificial intelligence-assisted approaches are beginning to be applied across the PC treatment pathway. Radiomics and deep-learning models trained on CT or MRI imaging have shown promising diagnostic and prognostic performance for detecting peritoneal metastases and predicting disease burden in OC, GC, and CRC [161,162]. Broader applications of AI to diagnosis, treatment planning, and recurrence prediction are also under active investigation [163]. While such tools remain at an early, largely retrospective stage of validation and have not yet been incorporated into prospective treatment-selection algorithms, they hold substantial potential to complement PCI- and biomarker-based selection with quantitative, reproducible risk stratification.

## 8. Methods

A review of recent scientific publications on the treatment of PC was conducted using the Scopus and PubMed databases, with no lower limit on publication year for the initial search. Among nearly 25,000 publications devoted to PC, studies investigating the potential use of IPC, HIPEC, PIPAC, ePIPAC, PDT, and PTT were selected, with particular attention to clinical trials and meta-analyses published between 2020 and 2026, in order to capture the most recent advances in intraperitoneal and physical treatment modalities. The search was performed using the following keywords: peritoneal carcinomatosis, intraperitoneal chemotherapy, hyperthermic intraperitoneal chemotherapy, pressurized intraperitoneal aerosol chemotherapy, electrostatic pressurized intraperitoneal aerosol chemotherapy, photodynamic therapy, and photothermal therapy. Following title and abstract screening, publications comprising randomized controlled trials, prospective and retrospective cohort studies, systematic reviews, meta-analyses, and key preclinical studies were selected for inclusion in this narrative review on the basis of their direct relevance to the diagnosis, treatment, and underlying mechanisms of PC.

## 9. Conclusions

The management of PC is undergoing a paradigm shift from a predominantly palliative approach toward an integrated, multimodal strategy. CRS combined with IPC, HIPEC, PIPAC, and ePIPAC has extended survival in selected patient populations beyond what systemic chemotherapy alone can achieve, while PDT and PTT offer mechanistically distinct locoregional alternatives with the potential for highly selective tumor cell destruction. Innovative approaches, including nanomedicine-based drug delivery, gene therapy vectors, photoimmunoconjugates, and combinations with immune checkpoint inhibitors, further expand the therapeutic landscape and may address the resistance mechanisms and limited tissue penetration that constrain current modalities.

Nevertheless, the evidence supporting each of these interventions remains heterogeneous and, in most cases, insufficiently robust for broad implementation in routine clinical practice. Patient selection, disease extent as quantified by the PCI, and prior therapeutic history critically determine the likelihood of benefit from any given intervention, making individualized management planning essential. Standardization of procedural protocols, development of validated biomarkers for patient stratification, and the conduct of large, adequately powered randomized controlled trials remain the principal prerequisites for translating promising experimental results into durable clinical benefit.

Looking forward, improving the precision, personalization, and reproducibility of locoregional therapies for PC will depend on a multi-faceted approach. Key developments include biomarker-guided patient selection, artificial intelligence-assisted diagnostic and treatment-planning tools, and synergistic combinations of regional therapy with systemic immunotherapy. Simultaneously, real-time intraoperative monitoring (such as optical dosimetry and fluorescence-guided imaging), alongside next-generation PSs and nanocarrier platforms, will further optimize local drug delivery and surgical accuracy. Ultimately, the convergence of these technological innovations with personalized molecular targeting holds the greatest promise for transforming patient outcomes and establishing a new standard of care in peritoneal surface oncology.

## Figures and Tables

**Table 1:** Outcomes of HIPEC in the treatment of OC.

Disease/Number of Patients	Prior Treatment	Main Outcome	Reference
OC/127	Neoadjuvant systemic chemotherapy	PFS was improved in favor of HIPEC (*p* < 0.001); OS improvement was also observed (*p* = 0.062)	[31]
Stage III and IV OC/71	Neoadjuvant systemic chemotherapy	RFS was improved in favor of CRS + HIPEC (*p* = 0.038); OS improvement was also observed	[25]
Stage III OC/584	None	3-year OS was improved in favor of CRS + HIPEC in cases of complete cytoreduction (*p* = 0.04); no difference in OS was observed in cases of incomplete cytoreduction (*p* = 0.19)	[32]
Stage III and IV OC/184	None	No significant differences in 5-year PFS (*p* = 0.569) or 5-year OS (*p* = 0.574) were observed between the HIPEC and control groups	[33]
Stage III OC/245	Neoadjuvant systemic chemotherapy; postoperative chemotherapy in both the HIPEC and control groups	Median RFS was 14.2 months in the CRS + HIPEC group and 10.7 months in the control group. Median OS was 45.7 and 33.9 months, respectively. At a median follow-up of 4.7 years, 62% of patients in the CRS group and 50% of those in the CRS + HIPEC group had died (*p* = 0.02)	[34]
Recurrent OC (FIGO IIIc and IV)/120	CRS, systemic chemotherapy; postoperative chemotherapy in both the HIPEC and control groups	3-year OS was improved in favor of CRS + HIPEC regardless of whether recurrence was platinum-resistant or platinum-sensitive (*p* < 0.01)	[35]
Recurrent OC/98	Chemotherapy administered 6–30 months before recurrence; postoperative chemotherapy in both the HIPEC and control groups	No significant differences in PFS (*p* = 0.05) or OS (*p* = 0.31) were observed between the HIPEC and control groups	[36]

Abbreviations: OS—overall survival; RFS—recurrence-free survival; PFS—progression-free survival; CRS—cytoreductive surgery.

**Table 2:** Comparative characteristics of HIPEC and PIPAC [34,81].

Parameter	HIPEC	PIPAC
Method of administration	Perfusion of a heated solution	Aerosol under pressure
Temperature	41–43 °C	37 °C
Most commonly used drugs	Cisplatin, oxaliplatin	Doxorubicin, cisplatin, oxaliplatin
Grade III–V complications	27–28%	4.6–9.2%
Advantages	High efficacy in macroscopic lesions	Lower toxicity, possibility of repeated procedures
Limitations	High complication rate, requires good performance status	Limited efficacy in nodules > 5 mm

**Table 3:** Results of PIPAC in patients with solid tumors.

Drugs	Number of Patients	Prior Systemic Treatment	Disease	Treatment Outcomes		Adverse Events	Reference
				Survival	Other Positive Effect		
Cisplatin + doxorubicin	15	3 lines of therapy	OC	Median PFS: 2.3 months (95% CI, 1.7–3.2); median OS: 17.1 months (95% CI, 5.6–not reached)	Radiological response: 26.7%; disease stabilization: 6.7%; partial histological response: 46.2%; overall intraperitoneal response: 30.8%	Two grade 3 complications: abdominal pain and anorexia; 14 grade 1 complications, most commonly abdominal pain, fatigue, and nausea	[94]
Doxorubicin + cisplatin	43	One or two prior lines of chemotherapy	Platinum-resistant recurrent OC	Median OS: 27 months (95% CI, 20.3–33.6); median PFS: 12 months (95% CI, 6.4–17.5); disease stabilization: 88.9%; progression: 11.1%	Pathological changes in biopsy specimens: minor response in 29.6%; absence of response in 70.4%	8.2% grade 2 adverse events (anemia, infection, etc.); 2% bowel perforation	[95]
Cisplatin + doxorubicin	99	On average, more than 2 lines of systemic therapy	Predominantly OC, other tumors of the female reproductive system	Median OS: 14.1 months; cumulative survival after 12 months: 56%, after 24 months: 28%	Tumor regression, 76%; improvement in PCI, 64%; reduction of ascites	Most adverse events were grade 1–2; grade 3–4 toxicity in 17% of patients; postoperative complications during combined CRS in 5 patients: perforation of the small bowel, perforation of the colon, insufficiency of the intestinal anastomosis, trocar-site metastases	[96]
Cisplatin + doxorubicin, oxaliplatin	20	One or more prior lines of chemotherapy	PDAC, CC	Median survival after the first PIPAC procedure was 9.7 months for PDAC and 10.9 months for CC	Tumor regression was observed in 50% of patients (62% in the cisplatin + doxorubicin group and 42% in the oxaliplatin group)	One intraoperative complication (small bowel perforation) and 18 postoperative adverse events of grade 1–2 severity	[97]
Cisplatin + doxorubicin	32	Most commonly systemic chemotherapy	GC	Median OS after diagnosis was 12.5 (95% CI, 10–17) months, and after the first PIPAC procedure: 5 (95% CI, 4–10) months	Reduction in median PCI from 10 to 7, *p* = 0.75; reduction in ascites volume (*p* = 0.56)	Intraoperative and postoperative complications (4.2%): severe neutropenia, intra-abdominal abscess, intraperitoneal bowel perforation	[98]
Cisplatin + doxorubicin, oxaliplatin, mitomycin C	31	Multiple prior lines of systemic therapy; colorectal/appendiceal tumors refractory to oxaliplatin	OC, LGSOC, colorectal/appendiceal cancer	OC: OS, 17.1 months (95% CI, 5.6–not reached); PFS, 2.3 months (95% CI, 1.7–3.2) LGSOC: OS: 11.6 months (range, 5.4–30.1); PFS: 4.3 months (range, 1.7–21.6) Colorectal/appendiceal cancer: OS: 12.0 months	Reduction in PCI: OC, 30.8%; LGSOC, 5%; PIPAC-MMC + FOLFIRI, 84% Histological response: OC, 46.2%; colorectal/appendiceal cancer, 58%; PIPAC-MMC + FOLFIRI, 79% Disease control rate: OC, 33.4%; LGSOC, 75%; colorectal/appendiceal cancer, 50%; PIPAC-MMC + FOLFIRI, 78.9%. Reduction in CEA level in the PIPAC-MMC + FOLFIRI group	No postoperative surgical complications were observed. Grade 3 toxicity was low (abdominal pain, anemia, anorexia), and no deaths were reported. Treatment completion rates were high: OC, 86.7%; LGSOC, 75%; colorectal/appendiceal cancer, 62.5%.	[99]
Cisplatin + doxorubicin	24	Systemic chemotherapy, 1–4 lines; gastrectomy in 50% of cases	GC	Median OS was 121 days (range, 66–625 days) after the first PIPAC procedure; in 8 patients who underwent more than 3 procedures, median OS was 450 days (range, 206–481 days) (*p* = 0.0376).	In 57% of cases, a decrease or stabilization of PCI was observed, as well as reduced or stable ascites volume	No severe chemotherapy-related toxicity; one postoperative complication	[100]
Cisplatin + doxorubicin	25	At least one prior line of chemotherapy; gastrectomy in 60% of cases; radiotherapy in 12%	GC	Median OS: 6.7 months	Disease control in 40% of cases; histological response in 36%; stabilization of ascites volume and PCI	Mild treatment-related toxicity was observed, including abdominal pain, nausea, elevated transaminase levels, hypoalbuminemia, and one case of subcutaneous emphysema	[101]
Cisplatin + doxorubicin	131	Exact data were not available	GC	Median OS was 11 months (range, 0–61 months) for the overall study population and 16 months (range, 2–61 months) for patients who underwent three or more PIPAC procedures	Significant or complete regression (PRGS 1/2) was observed in 73% of cases	Grade 3 toxicity was reported in 7 patients (4.9%), and grade 5 toxicity in 2 patients (1.4%)	[90]
Oxaliplatin ± systemic chemotherapy	24	Systemic chemotherapy in the majority of cases	CRC	Median OS after the first PIPAC session was 20.5 months (range, 0.13–34.7 months)	Tumor response according to PRGS was observed in 67% of patients; complete response in 21%; reduction in ascites volume	Two cases of severe postoperative complications (urosepsis and iatrogenic bowel perforation)	[102]
Oxaliplatin	77	Systemic chemotherapy and/or cytoreduction and HIPEC	Appendiceal cancer	Median OS after the first PIPAC session was 22 months (95% CI, 19.00–not reached)	Objective response in patients who underwent more than 3 procedures: disease control: 43%; PRGS 1–2: 49%; reduction in PCI: 37%	Severe adverse events (grade ≥ 3): 4.8%; intraoperative complications: 2.8%; postoperative complications: 4.9%	[103]

Abbreviations: LGSOC—low-grade serous ovarian carcinoma; PIPAC-MMC + FOLFIRI—PIPAC with mitomycin C combined with systemic FOLFIRI therapy in patients with colorectal cancer; OC—ovarian cancer; PDAC—pancreatic ductal adenocarcinoma; GC—gastric cancer; CC—cholangiocarcinoma; PFS—progression-free survival; OS—overall survival; PCI—peritoneal carcinomatosis index. Further studies involving repeated courses of PIPAC may facilitate the development of more effective treatment protocols for tumors with limited sensitivity to standard anticancer therapies. Further data will likely emerge from PICCOS, the largest randomized phase II clinical trial in the world, which is evaluating the efficacy of PIPAC in combination with systemic therapy in patients with CRC (*n* = 78) and GC (*n* = 72), and as an alternative to systemic therapy in patients with platinum-resistant OC (*n* = 66) [104]. The primary objective of the trial is to determine whether PIPAC improves progression-free survival compared with standard systemic therapy. PICCOS is expected to provide high-quality evidence regarding the efficacy of this approach, which is necessary for the potential incorporation of PIPAC into routine clinical practice. The principal limitations of the study are related to its open-label design, the heterogeneity of the patient population, and the different treatment regimens used across the study cohorts. Patient recruitment is currently ongoing, and the results have not yet been published.

**Table 4:** Results of clinical studies of PDT in patients with PC.

Disease	Number of Patients	Photosensitizer	PDT Parameters	Outcome	Toxicity	Reference
Colorectal carcinoma (*n* = 14); also included 3 patients with gastric carcinoma, gastric lymphoma, and benign diverticular disease	17	HpD, i.v. over 10 min, 48 h before surgery; 5 mg/kg (*n* = 2), 3 mg/kg or 111 mg/m^2^ (*n* = 15, reduced dose after early cutaneous phototoxicity)	510 nm (primary wavelength, large-surface adjuvant irradiation of the macroscopically normal tumour bed after resection, before anastomosis), using purpose-built uniform-dose delivery systems (IGLOO for flat surfaces, PELDS for the true pelvis, APERL for the perineal “tunnel” after abdominoperineal excision); energy density 32–62 J/cm^2^ depending on site; illumination 11–37.5 min; bare-fibre “top-up” to visible residual tumour in 2 patients (630 nm, 7–114 J/cm^2^)	Adjuvant (prophylactic) irradiation of the tumour bed, not treatment of gross disease. Among 9 patients with microscopic circumferential margin involvement, 8 had no local recurrence at 6–37 months (median 27); findings considered favorable versus historical local recurrence rates for this margin status, supporting a subsequent phase III trial	Cutaneous phototoxicity in 3/17 (1 mild, 1 moderate, 1 severe with blistering, at the higher 5 mg/kg dose); hyperpigmentation in 2; 3 anastomotic-area complications (subphrenic abscess, anastomotic breakdown, urethro-perineal fistula) and 1 perioperative death (myocardial infarction in a patient with pre-existing ischaemic heart disease)—all attributed by the authors to the extent of surgery rather than PDT; normal post-PDT ultrasound in all 9 irradiated ureters	[119]
Recurrent OC (*n* = 6) and uterine cancer (corpus, *n* = 1; cervix, *n* = 1)	8	m-THPC, 0.15 mg/kg i.v., 96 h before surgery/irradiation	652 nm (5 J/cm^2^), cylindrical diffusing fiber; intraoperative (laparotomy, *n* = 6) or laparoscopic (*n* = 2) delivery to resected areas and/or peritoneum	7/8 improved or unchanged on Karnofsky index; 6/8 remained relapse-free at last follow-up (7–32 months); mean tumor/fatty tissue m-THPC concentration ratio 4.5 (range 3.4–5.9)	No PDT-related systemic toxicity; mild cutaneous burns in 3 patients from inadequate light shielding; slightly increased postoperative ileus; 1 death from cardiac insufficiency/multiple organ failure (causal relation to PDT unclear); 1 death from ileus after later i.p. lavage (28 months post-PDT)	[120]
Intraperitoneal sarcomatosis (leiomyosarcoma, spindle cell sarcoma, synovial cell sarcoma)	11 (12 PDT treatments—one patient received repeat PDT after relapse)	Photofrin, 2.5 mg/kg i.v., ~48 h before surgery	532 nm green light (2.5 J/cm^2^, tissue penetration 3 mm)—mesentery, small bowel, large bowel; 630 nm red light (tissue penetration 5 mm)—5.0 J/cm^2^ (anterior stomach, omentum/duodenum/posterior stomach), 7.5 J/cm^2^ (diaphragm R/superior liver, diaphragm L/spleen, inferior liver), 10.0 J/cm^2^ (right/left peritoneal gutters, pelvis); boost 5–15 J/cm^2^ (630 nm) to gross residual disease. Real-time fluence monitored via 6 photodiodes (1 mobile + 5 fixed)	5/11 (45%) no evidence of disease at last follow-up (1.7–17.3 months, two confirmed by diagnostic laparoscopy); 6/11 (55%) developed intra-abdominal progression at 1–8 months; 2 deaths from progression (5.3 and 4.7 months); 1 patient achieved a second 6-month disease-free interval after repeat PDT for relapse	Universal systemic vascular/capillary leak requiring massive fluid resuscitation, with generalized edema peaking at 1–3 days post-PDT (worse with more extensive debulking); transient thrombocytopenia; elevated liver function tests; 1 postoperative pulmonary embolism complicated by ARDS	[121]
GI malignancies (*n* = 14: colon, appendiceal mucinous adenocarcinoma, gastric, pseudomyxoma peritonei, carcinoma of unknown primary, adenocarcinoid), ovarian cancer (*n* = 12, including primary peritoneal carcinoma), sarcoma (*n* = 15), peritoneal mesothelioma (*n* = 1)	42 (of 56 enrolled and 43 debulked; 1 excluded post hoc for benign pathology)	Photofrin, 2.5 mg/kg i.v., 2 days (48 h) before surgery	532 nm green light (2.5 J/cm^2^)—small bowel, bowel mesentery, colon (flat-cut fiber, mobile photodiode dosimetry); 630 nm red light delivered via fiber within intralipid-filled modified endotracheal tube (0.01% intralipid + Ca/Mg peritoneal fill)—5.0 J/cm^2^ (stomach), 7.5 J/cm^2^ (diaphragms, liver, spleen), 10.0 J/cm^2^ (pelvis, peritoneal gutters); focal boost doses (630 nm) to areas with severe tumor involvement; dosimetry via 5 fixed + 1 mobile photodiode	Median actuarial survival 21 months (all histologies combined; trend toward longest in ovarian, shortest in GI, not statistically significant); median time to recurrence 3 months (range 1–21); complete gross resection before PDT associated with significantly better survival than incomplete resection (log-rank *p* = 0.015). Retreatment with repeat PDT allowed for 3 patients with late recurrence	Universal capillary leak syndrome (15–20% body weight gain, ICU management, resolving in 3–7 days). Grade 3–5 toxicities: anemia 38%, LFT abnormalities 26%, GI toxicity (bowel obstruction etc.) 19%, hypotension 12%, edema/hyperglycemia/infection 10% each, hypocalcemia 7%; fistula 5% (*n* = 2), pulmonary embolism 2%, ARDS 5% (*n* = 2), hydronephrosis 2%. No bowel perforations (the dose-limiting toxicity in the preceding Phase I trial). 1 death (myocardial infarction, early in the trial, before enhanced cardiac pre-screening was implemented). No serious skin phototoxicity despite long photosensitizer half-life	[122]
OC (*n* = 33), gastrointestinal cancers (*n* = 37, including pseudomyxoma peritonei), sarcoma (*n* = 30)	100 enrolled (intent-to-treat); 71 received intraoperative light (23 ovarian, 22 GI, 26 sarcoma); 29 excluded intraoperatively (26 unresectable to ≤5 mm, 2 localized disease only, 1 benign pathology)	Photofrin, 2.5 mg/kg i.v., 48 h before surgery	532 nm green light (2.5 J/cm^2^)—mesentery, small/large bowel (flat-cut fiber); 630 nm red light via intralipid-filled endotracheal tube applicator—5.0 J/cm^2^ (stomach), 7.5 J/cm^2^ (diaphragms, liver, spleen), 10 J/cm^2^ (pelvis, abdominal gutters); boost up to 15 J/cm^2^ (630 nm) for gross disease (right diaphragm, gutters, pelvis); fixed + mobile photodiode dosimetry, power density ≤ 150 mW/cm^2^	All 100 patients progressed by analysis (median follow-up 51 months). Complete response at 6 months (laparoscopy/imaging): ovarian 3/33 (9.1%), GI 2/37 (5.4%), sarcoma 4/30 (13.3%). ITT median failure-free/overall surviva: ovarian 2.1/20.1 mo; GI 1.8/11.1 mo; sarcoma 3.7/21.9 mo (PDT-treated subset slightly higher: ovarian 3.0/22.0 mo; GI 3.3/13.2 mo; sarcoma 4.0/21.9 mo). Tumor Photofrin concentrations highly heterogeneous (inter- and intra-patient); tumor-to-normal bowel tissue ratio only 1.1–2.1, indicating limited selectivity	Near-universal capillary leak syndrome (median +20 L positive fluid balance in first 24 h, resuscitation needed 4–5 days). 2 deaths in immediate postoperative period (myocardial infarction; sepsis after reoperation for bleeding). ARDS/prolonged intubation (*n* = 4, infection or PE-related). Bowel fistula/anastomotic leak (*n* = 4). Wound complications (*n* = 4). Prolonged ileus/SBO (*n* = 3). Hydronephrosis (*n* = 9, grades 1–3). Mild cutaneous phototoxicity (*n* = 20, grades 1–2). Reversible hepatic/metabolic abnormalities common	[5]
Peritoneal pseudomyxoma (appendiceal mucinous carcinoma)	32 (vs. 25 HIPEC)	Photogem, 2.5 mg/kg i.v., 48 h before surgery; or Photosens, 0.2 mg/kg i.v., 2–8 h before irradiation	630 nm (Photogem) or 672 nm (Photosens); ~20 sequential overlapping fields (2.5 min each); fluence rate 6–10 J/cm^2^	Median survival 66 mo; 5-year OS 65.2% (vs. 86.6% HIPEC, with significantly higher baseline PCI and lower optimal cytoreduction rate in PDT group)	Complications in 9.4% (vs. 24% HIPEC); no perioperative mortality (vs. 12% HIPEC)	[123]
OC (high-grade serous carcinoma) with PC, complicated by small bowel obstruction and tumor necrosis	1 (case report)	Fotoran E6 (chlorin e6 derivative), 2.5 mg/kg i.v., infused over 30 min, 3 h before surgery	Fluorescence diagnosis: 405 nm (ALOD-01 laser) for intraoperative detection of residual tumor foci, used to guide extent of peritonectomy. PDT: 662 nm, low-intensity irradiation (power density 20 mW/cm^2^, energy density 10 J/cm^2^), applied to residual tumor after cytoreduction	FD identified occult tumor foci on the right diaphragmatic dome not visible macroscopically, leading to extension of diaphragmatic peritonectomy; histology confirmed adenocarcinoma at this site despite no visible metastatic change. Post-PDT histology of treated carcinomatosis nodules showed no residual adenocarcinoma foci on light microscopy. Staged approach: cytoreduction (CC0) + FD/PDT at stage 1, completion cytoreduction + HIPEC (mitomycin C, 44 °C, 60 min) at stage 2 (day 11). SF-36 quality of life improved progressively over 3 months (physical and psychological components both exceeding baseline by month 1–3). Disease-free at 4 months at time of writing	Right-sided hydrothorax requiring pleural puncture after stage 2; no PDT-specific complications reported; staged strategy adopted specifically to reduce perioperative risk given extensive surgery (posterior pelvic exenteration, right hemicolectomy, omentectomy, splenectomy, multi-quadrant peritonectomy	[124]

Abbreviations: PDT—photodynamic therapy; PC—peritoneal carcinomatosis; OC—ovarian cancer; OS—overall survival; FD—fluorescence diagnostics; HIPEC—hyperthermic intraperitoneal chemotherapy; LFT—liver function test; GI—gastrointestinal; ARDS—adult respiratory distress syndrome; LFT—liver function test; HpD—haematoporphyrin derivative; m-THPC—meso-tetrahydroxyphenylchlorin; ITT—intent-to-treat.

## Data Availability

The original contributions presented in this study are included in the article. Further inquiries can be directed to the corresponding author.

## Abbreviations

The following abbreviations are used in this manuscript:

PC	peritoneal carcinomatosis
CRS	cytoreductive surgery
HIPEC	hyperthermic intraperitoneal chemotherapy
PIPAC	pressurized intraperitoneal aerosol chemotherapy
ePIPAC	electrostatic precipitation pressurized intraperitoneal aerosol chemotherapy
IPC	intraperitoneal chemotherapy
PCI	peritoneal cancer index
OS	overall survival
PDT	photodynamic therapy
PTT	photothermal therapy
OC	ovarian cancer
CRC	colorectal cancer
GC	gastric cancer
PFS	progression-free survival
RFS	recurrence-free survival
LNPs	lipid nanoparticles
ROS	reactive oxygen species
PS	photosensitizer
mPDT	metronomic photodynamic therapy
PNPs	FRRG-doxorubicin nanoparticles
